# The Inequality Footprints of Nations: A Novel Approach to Quantitative Accounting of Income Inequality

**DOI:** 10.1371/journal.pone.0110881

**Published:** 2014-10-29

**Authors:** Ali Alsamawi, Joy Murray, Manfred Lenzen, Daniel Moran, Keiichiro Kanemoto

**Affiliations:** 1 Integrated Sustainability Analysis, School of Physics, University of Sydney, Sydney, New South Wales, Australia; 2 Programme for Industrial Ecology, Faculty of Engineering, Norwegian University of Science and Technology, Trondheim, Trondheim, Norway; 3 Institute of Decision Science for a Sustainable Society, Kyushu University, Fukuoka, Fukuoka, Japan; Cinvestav-Merida, Mexico

## Abstract

In this study we use economic input-output analysis to calculate the inequality footprint of nations. An inequality footprint shows the link that each country's domestic economic activity has to income distribution elsewhere in the world. To this end we use employment and household income accounts for 187 countries and an historical time series dating back to 1990. Our results show that in 2010, most developed countries had an inequality footprint that was higher than their within-country inequality, meaning that in order to support domestic lifestyles, these countries source imports from more unequal economies. Amongst exceptions are the United States and United Kingdom, which placed them on a par with many developing countries. Russia has a high within-country inequality nevertheless it has the lowest inequality footprint in the world, which is because of its trade connections with the Commonwealth of Independent States and Europe. Our findings show that the commodities that are inequality-intensive, such as electronic components, chemicals, fertilizers, minerals, and agricultural products often originate in developing countries characterized by high levels of inequality. Consumption of these commodities may implicate within-country inequality in both developing and developed countries.

## Introduction

There is no doubt that inequality in income exists both within countries and between countries. There also seems to be agreement that in both cases it is rising [51], [56]. The causes of this inequality are attributed variously to: conflict, governance and possession of natural resources [11], technological change [43]; jobs; transportation costs [31]; and globalisation [45], [19].

The UN sees inequality as a social justice issue as well as a threat to social, political and economic stability around the world [52], [55]. Recent studies also link inequality to environmental problems, sustainability, crime, disease and overall well-being which in turn is closely linked to life expectancy [21], [28], [56].

Attributing to inequality such dire global consequences as social, political and economic instability has implications for global action. Even though there are no simple solutions to what has been an intractable problem throughout history, our recognition of inequality and its consequences brings with it obligations. Competition for the consumer dollar has driven demand for ever-cheaper goods and services. Our demand sets in motion supply chains that ripple around the world. From time to time scandals erupt in the press, such as use of child labour in the making of a specific product [8], [16]. In such cases we take some responsibility for our consumption and pressure global businesses to improve their practice in third-world countries [5]. However, in the case of inequality existing within a particular country the relationship to exported goods is not so clear-cut. In consuming goods from this country – any goods – are we implicated in the inequality that exists in the exporting country? If yes, the simple solution would be to pressure the country to change through boycotting its goods just as we boycott firms for their use of child labour. However without our demand there would be no production and perhaps, as cited in the case of child labour, no jobs for those desperately in need of work [5]. Importing from unequitable countries could actually ameliorate the situation. In this study we ask simply: do more egalitarian countries import from less egalitarian ones? Dissecting the social impacts of a marginal dollar of trade with a country suffering from inequality is a difficult task. But regardless of whether trade between equal and less equal countries leads to a net gain or loss of welfare, understanding where inequality is occurring, who is benefiting from it, and which countries have the most polarized trade, provides information useful for understanding the dynamics of inequality and trade.

This study introduces the novel concept of an ‘inequality footprint’, which is defined as the Gini index of the workforce that is directly and indirectly required to satisfy the consumption of a given population. Thus, the inequality footprint extends beyond the boundaries of a particular country and includes people working in countries that produce goods and services bound for international trade. In this work we undertake a quantitative analysis of the inequality footprint of the world's nations and portray these footprints using a number of intuitive measures. We do not argue whether trade between more and less equal countries is a social good or ill, but merely provide a robust, global, account of inequality and trade upon which such further economic analysis may be built.

In section 2 we provide a context for our work through an overview of the inequality literature, discussing the issue from the perspective of world bodies such as the UN and the IMF and drawing on some of the within-country inequality literature. Following that we provide our methodology and our data sources in section 3. We present the results in section 4 and section 5 concludes.

## Background

### 2.1 Inequality as a global issue

The first target of the first millennium development goal (MDG1) is to halve, between 1990 and 2015, the proportion of people whose income is less than $1 a day (http://www.un.org/millenniumgoals/poverty.shtml accessed 01/10/12). Growing inequality makes it harder to reach this goal. As Kofi Annan observed in 2005: “we cannot advance the development agenda without addressing the challenges of inequality within and between countries” [52]. Over the past 20 years inequality has risen within more countries than it has fallen [57]. In 2007 the UN News Centre reported that the United Nations Assistant Secretary-General for Economic Development, Jomo Kwame Sundaram, was concerned about a significant and disturbing increase in inequality within and between countries around the world, which he attributed to a worldwide decline in the number of jobs (http://www.un.org/apps/news/story.asp?NewsID=21526&Cr=globalization&Cr1#.UF9-vRiTbbs accessed 24/09/12). Even in countries in Northern Europe that have the world's lowest income inequalities [26], such as Germany, Denmark and Sweden [43] inequality grew in the 2000s probably because of increased income disparity [13].

Atkinson, Piketty, and Saez [3] see increasing income disparity as at least partly attributable to an unprecedented surge in top wage incomes [44]. They attribute the evolution of the top one per cent share of wealth in various countries to political changes (e.g. Reagan in the US and Thatcher in the UK), wars, financial crises, global factors and taxation. Whatever the reason the wealthiest 20 per cent now account for 86 per cent of all private consumption and the poorest account for around one per cent [55]. Even in China and Russia increases in income are invariably going to the top one per cent of the population [3], [12]. This matters because inequality within countries affects people's well-being [28], [56]; it breeds social resentment and generates political instability as people feel that they are losing out while others are becoming rich [43], [10]. Such inequality would seem to be a recipe for disaster.

### 2.2 Within country inequality

In the past it was assumed that globalisation would raise the income of almost all nations and help redress inequality [31]. However many studies have found no obvious relationship between a country's participation in globalisation and positive changes in inequality within countries [13], [26]. Although a study conducted by the IMF (2009:35) shows inequality in Mexico fell after the mid-1990s [19] other studies disagree. A 2008 study of wage inequality in Mexico found increased access to export markets led to growing wage inequality [58] with the need to produce better products for export than for domestic markets within industries and hence the need for upgrading of skills for some workers. Thus wage disparity increased within manufacturing industries where smaller less productive plants with less skilled labour supplied local markets while larger more productive plants with highly skilled labour produced goods for export. Other studies quoted by Pavcnik [45] found a similar process in Argentina and Indonesia [12], [52].

A related skill-based driver of wage inequality has been a general increase in use of information and communication technologies (ICT) by some industries, which has increased their demand for high-skilled, college educated, workers [45], [26]. Where access to technology skills and education are available only to the elite the poor have no ability to move from unskilled to higher skilled occupations (e.g. from agriculture to industry) thus increasing inequality. This selective apportioning of education is not confined to developing countries. A 2005 UN study found some of the highest income inequalities – as well as in Mexico and Turkey – were in the USA [52] much of which in the US was attributed to access to education, in particular skill-biased technological change (SBTC) [17], [6]. Coincidentally the life expectancy of poorly educated women in the US has also slipped in the last decade from a gap of 5.8 years to a gap of 8.6 years compared with women with a Bachelor's degree or higher [42]. Equal access to education, job creation and skill development opportunities are consistently seen to be the most important factors in the building of a more equitable society [43], [45].

The issue around access to education and its effect on inequality is not confined to the production of finished goods. Goldberg [18] suggest that global trade in intermediate goods also has an effect on wage inequality with some large firms from developed countries locating unskilled parts of production in developing countries and reserving any skilled part of production for developed countries [45]. Apart from relieving firms of the need to develop skills in workers in developing countries, this can also have the effect of firms shifting responsibility for wages and working conditions away from the regimes of developed countries where conditions are generally governed by industrial law to countries where such laws do not exist or else are only just emerging. In this case (i.e. production of intermediate goods) and in the case of final products the producer has some control over skill development, working conditions and wages and can act to alleviate inequality if motivated to do so.

At the other end of the supply chain consumers have some control over the demand for imported goods. For example consumers in Scandinavia live in some of the most equal of all OECD countries [44] and amongst the most equal in the world [26] yet they import goods from some of the most unequal. One such is China where although foreign demand has increased employment considerably [26] it has induced jobs mainly in low-paid, low and medium-skilled areas without generating new job opportunities for the growing number of college-educated workers in the middle thus adding to inequality [38]. Another Scandinavian trading partner, the Russian Federation, also has high inequality [50]. The economic recessions in Asia, Latin America and the Russian Federation following the financial crises of the 1990s brought increased unemployment and inequality, both of which contributed to the erosion of social cohesion. This was especially true of Russia, where the Gini inequality index rose from 0.397 in 2001 to 0.422 in 2009 [12] and which recorded one of the lowest life satisfaction scores in the world just above Moldova, Ukraine and Belarus [28]. Other Scandinavian trading partners such as Brazil and the Philippines are experiencing worsening inequality, which has been linked to trade liberalisation. For example a 2005 UN study [50] found that trade liberalisation caused wages to decline in Brazil and Mexico especially in the case of unskilled workers. The study went on to also link trade liberalisation with widening inequality in the Philippines and Eastern Europe.

### 2.3 Responsibility

The debate around the causes of within-country inequality, and even within which countries it is rising or falling, is unresolved [4]. Also different methodologies for studying inequality can produce different results [39] making it difficult to generalise causes and specific effects. So who may be held responsible for improving the situation? Blaming bad government in such countries [11] is to oversimplify the issues. After all, global companies do business with these countries. As Goldberg [18] point out, increasingly global trade is about movement of goods between firms located in different countries rather than the flow of goods between countries, so perhaps such firms should take the blame for supporting – and possibly exploiting – inequality. Blaming the final consumer for demanding goods contaminated by the implication that they arose from countries of gross inequality is also a simplification. Consumers could also be seen as providing jobs for the desperately poor. People need to work and often a poorly paid job in a country of high wage disparity is better than no job. Firms, governments and consumers all play a part in the complex web of production, wages and work. With a better understanding of within-country inequality all can play a part in bringing about a more equal society.

The ‘inequality footprint’ can provide a tool to assist in tracking the inequality implicated in goods as they move around the world. The concept of ‘footprint’ is familiar from the literature. Studies have been conducted, for example, on the emissions footprint showing that consumption in one country can cause emissions in another [59]. Or the water footprint that can track embodied water in goods as they move around the world and can distinguish ‘scarce water’ inputs from non-scarce water inputs [22]. We also know that consumption in one country requires the input of labour from other countries [38], which may or may not be problematic depending on work conditions and the age of workers. Just as campaigns for fair and ethical work conditions that distinguish unethical (e.g. child labour) from other labour we distinguish problematic (unequal) labour from equal labour. We define an inequality footprint as the Gini index of the workforce that is directly and indirectly required to satisfy the consumption of a given population. In this way we cast income inequality into the same footprint accounting framework as scarce water, GHG emissions and unethical work practices. These accounts may then be used to study the correlative or causal relationship between consumption and inequality.

## Methodology and Data Sources

### 3.1 Data sources

This section describes the data foundation of this work. We use three databases covering 187 countries:

1. Employment: the International Labour Organization's LABORSTA database [24], and the United Nations System of National Account UNSNA-Official Country database [53];
2. Income and global inter-industry transactions data: the Eora Multi-Regional Input-Output (MRIO) database [14], [32], [33];
3. Gini index: the Standardized World Income Inequality Database (SWIID) version 3.1 [47], and the Organization for Economic Co-operation and Development (OCED) database (http://stats.oecd.org/) provide Gini indices referring to both before-tax and after-tax income. In this paper we have used the after-tax Gini index data. Also we including Gini index database from the World Bank (http://data.worldbank.org/).

In essence, we use data on employment, income and Gini indices to construct income distributions for the 187 countries in our study (see Appendix S1 in File S1). These income distributions are then cast into the shape of a so-called national satellite account (see Appendix S3 in File S1) that accompanies global inter-industry (MRIO) transactions data. In unison, the MRIO data and income distribution satellite enable tracing economic activity in one location to income distributions in other locations around the world. This is explained in detail in the remainder of this section.

### 3.2 Basic input-output theory

This work is concerned with enumerating employment and inequality footprints for the world's economies. As in previous studies on carbon footprints [20], [46], water footprints [15], and biodiversity footprints [34], we apply the method of economic input-output (IO) analysis [36].

The centrepiece of any IO analysis is an assembly of three matrices: one *N*×*N* intermediate transactions matrix **T** with elements *T_ij_* that represent monetary amounts intermediate demand from supplying economic sectors such as agriculture, forestry, fishing, mining, manufacturing, utilities, trade, transport, or services *i* = 1,…,*N* into using sectors *j* = 1,…,*N*; one *K*×*N* value added matrix **v** with elements *v_kj_* that represent monetary amounts of primary input from value-added categories i.e. wages and salaries, gross operating surplus, and net taxes on production *k* = 1,…,*K* into using sectors *j* = 1,…,*N*; and one *N*×*M* final demand matrix **y** with elements *y_jm_* that represent monetary amounts of final demand from supplying economic sectors *i* = 1,…,*N* into final demand categories i.e. household consumption, government final consumption, gross fixed capital expenditure, and changes in inventories *m* = 1,…,*M*.

This assembly is a balanced financial account in a sense that total uses **x** = **T1** *^N^*+**y1** *^M^* equal total supply **x**′ = **1** *^K^* **v**+**1** *^N^* **T**, where **1** *^N^* =  {1,1,…,1} etc are suitable summation operators, and where the prime (‘) symbol denotes transposition. Setting **T1** *^N^* = **Ax**, we find the fundamental input-output identity **x** = **Ax**+**y1** *^M^*, where **I** is a *N*×*N* identity matrix, **A** is the input coefficients matrix, and is the famous Leontief inverse.

### 3.3 Extended input-output analysis

It was always Leontief's intention to put IO analysis to use for solving environmental and social problems [35], [36]. To this end, the input-output account assembly (**T**, **v**, **y**) is supplemented by a 1×*N* environmental or social satellite account **Q** with elements *Q*_1*j*_ describing amounts of some environmental or social variable (for example energy, emissions, employment) associated with (used by, emitted by) economic sectors *i* = 1,…,*N*. Setting **Q1** *^N^* = **qx**, we find environmental or social accounting identities as *Q* = **Q1** *^N^* = **q**, where *Q* represents the economy-wide total of the satellite account **Q**. The vector **q** holds so-called intensity coefficients (for example energy intensities, employment intensities) that describe the amount of the satellite variable associated with (used, emitted per) one unit of total use. In contrast, the multiplier **m** describes the amount of the satellite variable associated with one unit of final demand. The multiplier **m** includes all direct and indirect flow-on effects rippling throughout the complex supply-chain network of the entire economy, as described by the Leontief inverse **L**, where **m** = **qL**. The environmental and social accounting identities are the basis of the widely used Leontief demand-pull model, which interprets *Q* as the total environmental or social inputs required to satisfy a final demand bundle **y**. Therefore, *Q* is also referred to as an environmental or social footprint (depending on the nature of the satellite variable).

Whilst emissions satellite accounts are the basis of many carbon footprint studies [20], [46], the satellite accounts **Q** used in this work are employment accounts **E** in units of full-time equivalent person-years, and wage and salary income accounts **W** in units of US$. These accounts were constructed at a detail of 187 countries (see Appendix S1 in File S1) with a combined 15,909 sectors in order to complement a matching Multi-Region Input-Output (MRIO) framework (**T**, **v**, **y**) of the world economy [33], [37], [40], [41]. Whilst the income satellite account **W** is based on a multitude of data sources [33], the employment satellite account **E** is based predominantly on data published by the ILO [24]. Further details on the construction procedures followed are given in Appendix S3 in File S1.

### 3.4 Inequality satellite accounts

The employment and income accounts **E** and **W** can be used to determine employment and income footprints of nations [1], [38]. In order to construct satellite accounts on income *inequality*, data on employment and income have to be collected, and assembled into a separate satellite account, for a number of income classes. Such detailed data are hard to come by for most countries as a whole, for example the Household Expenditure Surveys of Brazil [23], and Australia [2], let alone for a complete suite of world nations and broken down into economic sectors as represented in the MRIO database that we use. We have therefore devised a strategy to estimate distributions of income across *C* income classes for individual industry sectors in individual countries from three data items: a) the country's Gini index γ, b) the sector's total income payments for employees *I*_tot_, and c) the sector's total workforce *P*_tot_.

**Figure 1:**
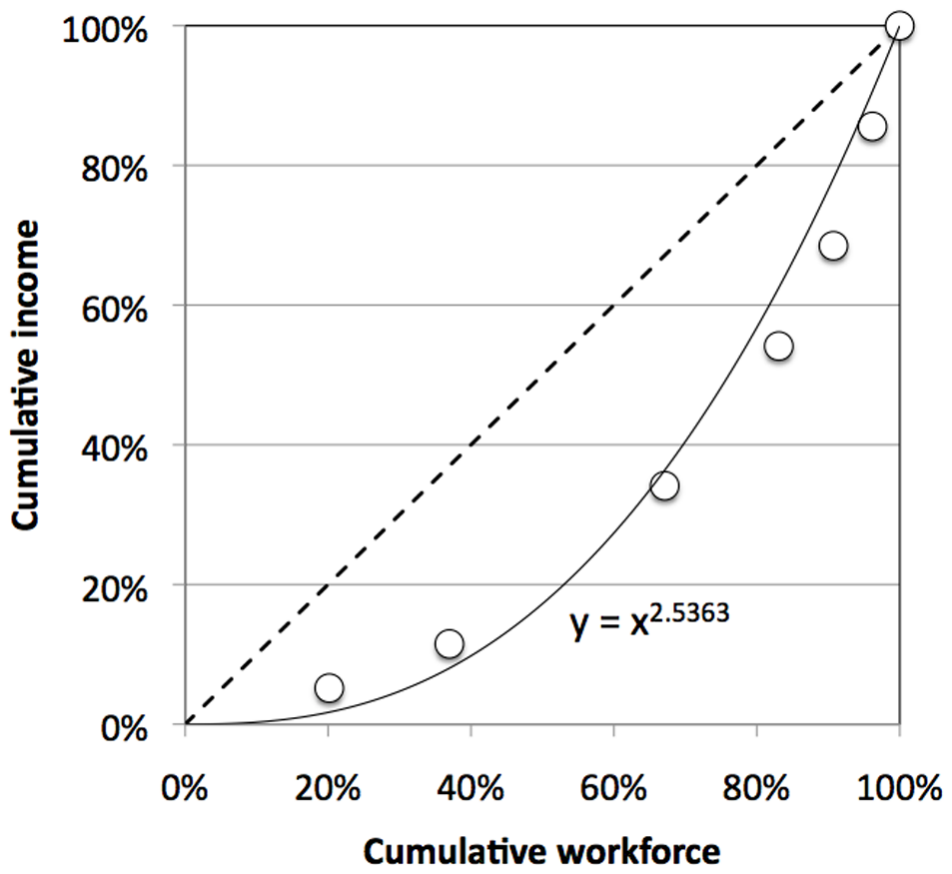
Lorenz Curve for Brazil’s Income Distribution in 2009. *Notes:* Circles: data from IBGE [23]; solid line and regression equation: power function approximation from Eq. 3; dashed diagonal line: Lorenz curve for complete income equality. Further examples about the quality of the power function fit can be found in Fig. S1.



Income inequality is typically depicted in Lorenz curves (Fig. 1). Cumulative income *I*(*c*) at income class *c* (normalised to 100%)
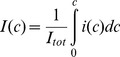
(1)is plotted against cumulative workforce (equally normalised).

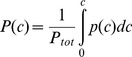
(2)


In the case of perfect income equality, cumulative income increases proportionally with cumulative workforce, and the Lorenz curve is a diagonal connecting the origin with the point {1,1} (dashed line). Any degree of income inequality will see cumulative workforce increase more rapidly than cumulative income, thus leading to a convex Lorenz curve (circle markers in Fig. 1) 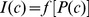
(3)


This Lorenz curve can be approximated by a power function (solid line in Fig. 1)
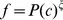
(4)


Total income equality corresponds to 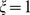, and 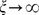 leads to increasing inequality. A power function also ensures that all Lorenz curve representations pass through the origin {0,0} and the point {1,1}.

The Gini inequality index 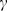 can be calculated as the ratio of the area between the diagonal and the Lorenz curve, and the area below the diagonal:
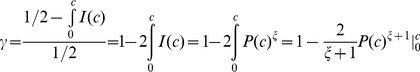
(5)


Since *P*(0)  = 0 and *P*(*C*)  = 1 according to the definition in Eq. 2, we can evaluate the integral, and find the relationship between 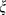 and 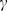 as
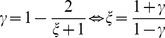
(6)


Fig. 1 shows an example for an approximation of a Brazilian Lorenz curve with 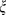 = 2.51 (solid line), based on data (circles) yielding 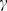  = 0.43. We have added evidence in support of the power function approximation in Fig. S1.

Inserting Eq. 6 into Eq. 3, we find the income distribution
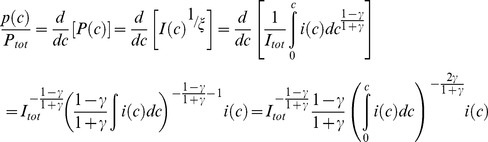
(7)


**Figure 2:**
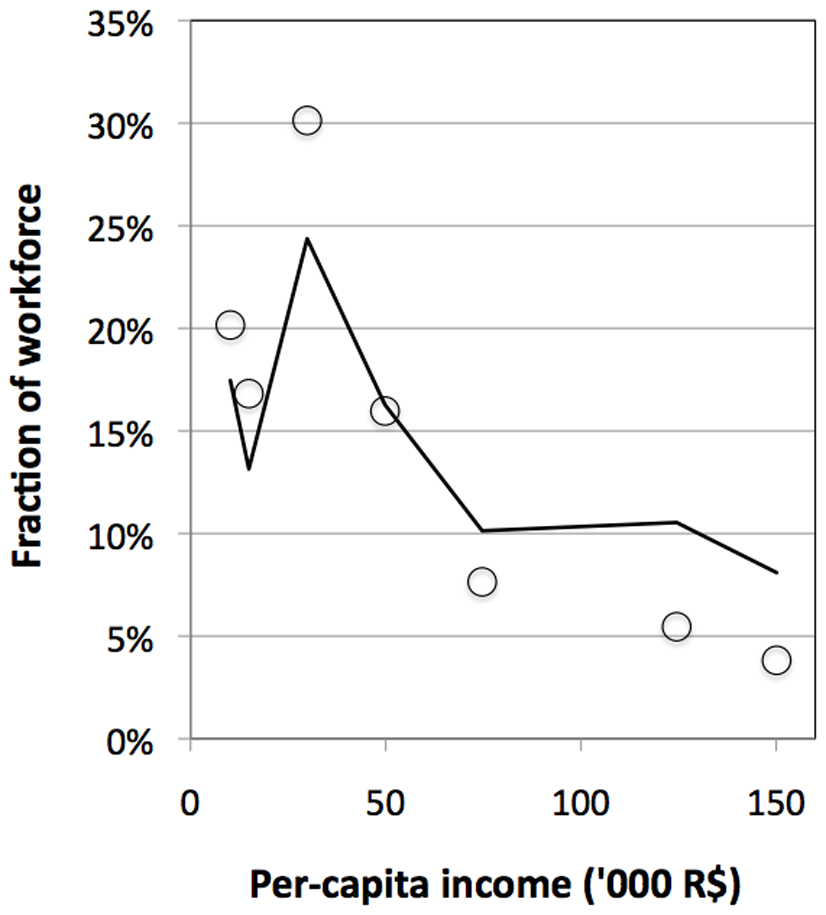
Income Distribution for Brazil in 2009. *Notes:* Circles: data from IBGE [23]; solid line: power function approximation from Eq. 7.

Eq. 7 can be evaluated by choosing income intervals *i*(*c*), and calculating corresponding workforce fractions. Choosing a large number *C* of intervals will increase the resolution of the approximation. Fig. 2 shows an example for an approximation of a Brazilian Lorenz income distribution curve, based on data (circles) yielding 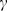 = 0.43.

The average wage *w*(*c*) for each income class *c* can be determined via
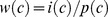
(8)


Assuming that an income class satellite would identify *L* « *C* classes according to wage intervals *w_l_*<*w*(*c*) ≤w*_l_*_+1_, and assuming that one satellite class *l* ∈ {1,…,*L*} contains *n_l_* such wage classes {*c*_1,*…,nl*_}, the income class satellite account **Q** simply reads
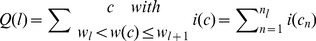
(9)


The workforce in each satellite class *l* is then taken from Eq. 7 as
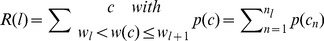
(10)


The average wage in each satellite class *l* is 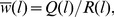
(11)with 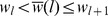. Repeated for all sectors in the MRIO database, this procedure will yield two satellite accounts **Q** and **R** sized *C*×*N*, one for employment by class, and one for income by class.

### 3.5 Inequality footprints

Inequality footprints of nations can now be calculated subjecting the income and employment class accounts to the Leontief-type demand-pull terms of income inequality would then proceed as follows. Assume a *N*×1 vector 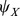 describing a final demand bundle originating from country *X*, the inequality footprint of which is to be evaluated. The impact of 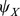 in terms of income earned in economic sectors *j* in various classes *c* is 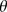 =  {*θ*_χφ_}  = 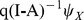, where 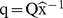 holds income by sector and by class per unit of total use, and where 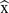 is a diagonal matrix holding total use. The impact of 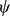
in terms of employment in various classes is 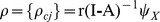, where 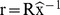 holds employment by sector and by class per unit of total use. In other words, 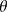 and 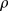 are the income and the workforce, respectively, broken down by economic sector and by class, that are required directly and indirectly to satisfy the final demand bundle 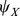, or in other words the *income and employment footprints* of the final demand bundle 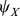. The average wage 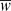 paid to this workforce in sector *j* is 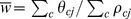.

The Gini index γ characterising this global workforce is 
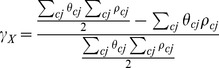
(12)



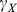
 includes people working in country *X* ανδ in other countries (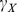 refers to the inequality within the entire world, and therefore relates to the between-country perspective of inequality described by the ‘World Inequality’ concept in Milanovic [39]. In general the more unequal the combination of import origins, the higher the global inequality footprint. This perspective is equivalent to Milanovic [39] also offers a method for decomposing global inequality into within-country, between-country, and overlapping effects). It is possible to isolate the inequality effect of the domestic final demand bundle 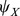 on particular countries 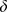 by only considering the income and workforce needed from the sectors j 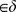 belonging to those countries 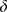:
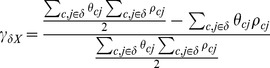
(13)


The *inequality footprint* of a nation can be defined as a weighted sum over countries of production origin
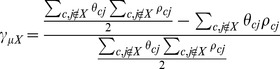
(14)


The *inequality footprint of imports* can be defined as a weighted sum over countries of imports origin
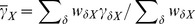
(15)


The origin country weights 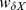 could be set to the income footprints 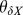 or employment footprints 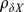, or to the product 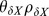 of both (compare with Part A in Eq. 1 in [39]). In general the more unequal the selection of import origins, the higher the inequality footprint of imports.

### 3.6 Qualifications

To our knowledge this is the first time that an income inequality indicator (through the Gini index measure) has been combined with an input-output calculus. Nevertheless, the implementation of the method we have so far described has a number of shortcomings.

First, as we described above, income distributions are largely unavailable for most countries, and therefore we needed to construct income distributions from Gini indices by fitting power functions. These power function fits will necessarily deviate from “true” income distributions, but as we have shown in the example for Brazil, and in more examples listed in Fig. S1, such deviations are likely to be small.

Second, Gini indices are not available as continuous time series for all countries. In the absence of continuous information, we interpolated the Gini indices for missing years on the basis of those for neighbouring years. We treat missing values at the beginning and the end of our period of analysis by setting them equal to the data available for the closest year. Large gaps exist only for countries with a combined small fraction of GDP, and associated errors are probably small. For Gini index availability see Appendix S2 in File S1.

Third, data for Gini indices, income distributions, or Lorenz curves are almost impossible to locate for individual industry sectors, therefore we needed to assume that the national Gini index applies to all sectors of the economy. This procedure will lead to allocation errors in cases where inequality varies significantly between sectors, *and* where a country predominantly exports those products made by sectors with significantly above- or below-average inequality (for example, soybean in Brazil as opposed to other sectors such as petroleum oils).

## Results

**Figure 3:**
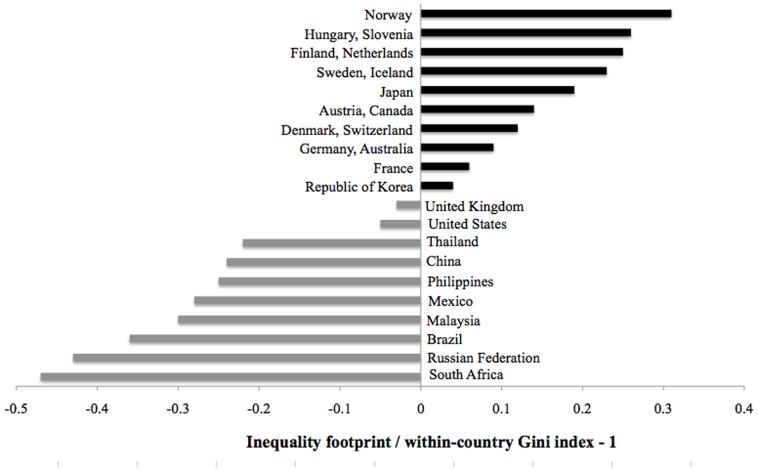
The World's Top and Bottom Countries in Terms of the Disparity between their Inequality Footprint and Within-Country Inequality. *Notes*: The horizontal axis depicts the departure from 1 of the ratio between inequality footprint and within-country Gini index.

We find that the inequality footprint of countries differs substantially from their within-country inequality (Gini index) (Fig. 3). Countries occupy top ranks in Fig. 3 if their inequality footprint is significantly larger than their within-country inequality. This can be either because their imports come from unequal countries (for example Japan which imports from China, Thailand, Russia, etc see Table 1), or their own country's society is very equal (for example Sweden which occupies a rank similar to that of Japan but for different reasons see Table S6 in File S1). The opposite pattern holds for the bottom-ranked countries. Their inequality footprint is significantly smaller than their within-country Gini index. That means either their imports come from equal countries (for example in the case of Russia), or their own country's society is very unequal (for example in the case of South Africa) (see Table S6 in File S1). In addition to top- and bottom-ranked countries we include France, Korea, United Kingdom, United States, Thailand, and China because of their significant share of the world's Gross Domestic Product (GDP) (for more information about rankings see Table S5 in File S1).

**Table 1:** This Table Ranked List of Countries as in Fig. 3 but with Detail on Inequality-Implicated Commodities and the Labour Embodied in Imports from Countries that have a Gini Index above 0.4.

Country	% Above 0.40	Inequality-implicated commodities (country embodied labour (‘000 FTE) implicated-commodity)	% 0.35–0.4	% 0.3–0.35	% Less 0.3
Norway	34	CHN 128 mob; RUS 82 po; THA 75 tel; PHL 40 ec; BRA 38 sb; MDG 20 va	16	27	23
Slovenia	33	RUS 43 ng; CHN 26 mob; THA 13 ac	13	31	23
Hungary	38	RUS 157 po, gas; CHN 70 mob; THA 27 ec	10	23	28
Finland	43	RUS 195 po; CHN 86 pt, mob; THA 45 tra	13	20	24
Netherlands	36	CHN 2,150 mob; RUS 252 po; THA 202 mob; BRA 182 sb; PHL 175 ec; MDG 78 ff; UGA 70 tr	20	25	19
Sweden	29	RUS 145 po; CHN 135 mob; THA 80 mob; PHL 45 ec	16	24	31
Iceland	39	RUS 11 po; THA 9 dum; CHN 8.8 com	18	22	20
Japan	51	CHN 21,500 mob; THA 2,360 med; RUS 2,134 po; PHL 2,057 ec; MDG 1,161 va; MYS 728 ch; BRA 500 cop; LKA 418 sf	23	19	6
Switzerland	55	RUS 3,000 po, jew; PHL 668 ec; CHN 407 com; THA 379 cw; LKA 323 jew; ECU 266 coc, ros; UGA 245 tob; MDG 145 coc	15	15	15
Austria	30	CHN 155 mob; RUS 139 po; THA 73 wd; MDG 69 ff, va; PHL 50 ec; BRA 39 ff	15	23	32
Denmark	32	CHN 125 com; RUS 101 po; THA 70 mt	16	26	27
Canada	39	CHN 3,251 mob; PHL 295 tx; MEX 275 gen; RUS 174 po; THA 180 mob; MDG 144 va; BRA 147 med, tra	35	16	9
Germany	43	CHN 8,490 mob, clo; RUS 3,184 po; MDG 683 va; BRA 605 pu; PHL 575 ec, sc; THA 591 jew, mob	15	25	17
Australia	42	CHN 4,884 com, med; THA 488 med, mob; MYS 313 scr; PHL 364 ec; PNG 120 po; RUS 122 po; BRA 108 io	23	27	9
France	38	CHN 4,894 com, pm; MDG 1,173 ff, va; RUS 776 po; MAR 608 clo, veg; BRA 366 pu; THA 360 ac	20	27	15
Republic of Korea	41	CHN 974 mob, pm, clo; PHL 381 ec, ff; THA 377 tel; RUS 298 po, coal; MYS 155 ec	25	20	14
United Kingdom	38	CHN 7,284 mob, tel; RUS 896 po; THA 879 ec; PHL 820 ec; MYS 237 tel	20	23	20
United States	55	CHN 27,178 tra, rubb, med,clo; MEX 4,853 mot, cof; PHL 2,010 ec; RUS 1,428 po, jew; BRA 1,345 tra, tob; THA 1,368 tel, jew; MDG896 va	10	17	18
Thailand	27	CHN 261 com, mob; PHL 130 ec, tra; RUS 95 po, pu; MYS 105 ec;	40	19	13
China	32	THA 1,129 rubb, mob; RUS 1,040 wd, io; MDG 720 wd; PHL 741 ec; MYS 463 wd, rubb, ec; BRA 313 io, po;	25	26	18
Philippines	30	THA 149 rc; CHN 69 pg; RUS 34 io, po; MYS 23 ec, ec	31	26	13
Mexico	41	CHN 281 com; RUS 172 po; BRA 157 tra; THA 98 pt; GTM 83 sug; PHL 78 ec; CHL 77 ff; MDG 75 nb; MYS 41 ec	34	16	10
Malaysia	38	THA 638 rubb, mob; CHN 289 ec; PHL 227 ec; RUS 101 io, po; MDG 49 clov	29	24	9
Brazil	52	RUS 585 po; CHN 413 tel; ARG 362 mot; BOL 349 pg; THA 178 med, tra; PRY 163 ma; PHL 166 ec; MEX 111 ref; VEN 101 po, coke	18	17	13
Russia Federation	12	UKR 2,293 rw,io; BLR 1,180 n.e.s.; TJK 595 cot, ff; KAZ 530 io; AZE 309 ff; LTU 52 dp,ff	18	20	50
South Africa	44	CHN 129 com, mob; ZMB 126 cot, cop; THA 72 rc; RUS 55 cop; BRA 38 mv; MDG 36 clov	20	20	16

*Notes*: The inequality footprint is broken down into contributions from trade partners with a Gini index of above 0.4, 0.35–0.4, 0.3–0.35, and less 0.3. ARG Argentina, AZE Azerbaijan, BLR Belarus, BOL Bolivia, BRA Brazil, CHL Chile, CHN China, ECU Ecuador, KAZ Kazakhstan, LKA Sri Lanka, LTU Lithuania, MDG Madagascar, MEX Mexico, MYS Malaysia, PHL Philippines, PRY Paraguay, RUS Russia, THA Thailand, TJK Tajikistan, UGA Uganda, UKR Ukraine, VEN Venezuela, ZMB Zambia, ac air conditioner, ch wood charcoal, clo clothes, clov cloves, coc cocoa, cof coffee, cop copper, cot cotton, cw clocks and watches parts, dp dairy products, dum dumpers, ec electronic circuits, ff fresh fruits and juices, gen electric generators, io iron ores, jew jewellery, ma maize, med medical articles and instruments, mob mobile, mot electric motors and it's parts, mt canning meat, n.e.s. not elsewhere specified, nb niobium ore, ng natural gas, pg petroleum gas, pm printing machine, po petroleum oil, pt part of telephone, pu chemical wood pulp, rc milling rice, ref refrigerators, ros roses, rubb natural rubber, rw railway parts, sb soya bean, sc solar cell, scr monitors and projectors, sf seafood, sug cane or beet sugar, tel telephone, tob tobacco, tr live tree, tra tractors parts and accessories, tx textiles, va vanilla, veg vegetables, wd wood in rough.

Scandinavian countries have the largest disparity between their inequality footprint and their within-country inequality (Fig. 3 and Table S6 in File S1). Further investigation of the commodities that are being traded reveals that inequality is often hidden from the final consumer because of complex supply chains that stretch across multiple countries and producers. The supply chains ending in electronic goods often originate in export-oriented industries in developing countries producing inputs such as electronic components, chemicals, fertilizers, minerals, and agricultural commodities characterised by high levels of inequality (Table 1). For example, the consumption of a mobile phone in Norway may require labour from China and Thailand to assemble phone components, which in turn requires electronic circuit manufacturing in the Philippines and Malaysia, which in turn relies on Russian petroleum oils. Despite the origins of Scandinavian imports often being unequal societies that does not mean the consumption in Norway, for example *causes* inequality in another country. It could even be that the consumption decreases inequality. We may simply say that through their consumption Norwegians are *implicated* in or associated with the situation of unequal income distributions (compare Section 2.3). Although Norwegian society itself maintains high levels of equality through active pursuit of re-distributive tax, unemployment and social benefits policies, employment protection legislation for workers, and reduced taxes on labour for low-income workers [44].

Many developed countries have an inequality footprint that is higher than their within-country inequality largely because of their imports from more unequal developing or transition economies. Notable exceptions are the United States and the United Kingdom (Fig. 3). These exceptions are due to their own country's society being unequal (see Table S6 in File S1). Whilst their inequality footprint is not much different from that of Switzerland or the Netherlands, their own Gini indices of 0.33 and 0.36 are considerably above those of most European countries (Gini index <0.30). Seventy million full-time equivalent workers from outside the USA support the lifestyle of US citizens (this is equivalent to 50% of the total workforce in the USA for 2010), approximately 40 million of them are from countries with high inequality (see examples in Table 1). The main imports of developed countries (for example US) are electronics and clothes, raw materials and natural resources, and manufactured parts that are used as an intermediate input into making other products (Table 1). Examples for the latter category are: motor vehicle parts from Mexico (where about 5 million laborers are working for US) exported for input into car production in the US; electronics from China (about 27 million working for US); electronic integrated circuits from the Philippines (about 2 million working for US) which are also used as an input into electronic production in the US. Despite the high amount of labour embodied in imports from unequal countries and a large inequality footprint, the within-country Gini index of the United States is higher than its total inequality footprint. Some economists attribute [9] the rising income inequality in the US to the large gap in wages between college-educated and high school workers. Another factor affecting increasing inequality in the US is the large disparity in income distribution between skilled and unskilled workers and the low level of income redistribution. Further, the opening up of trade in the United States could affect inequality, for example through the North American Free Trade Agreement (NAFTA) between the United States, Canada, and Mexico in 1994. This treaty increased income inequality in the US because the unemployment rate increased when unskilled jobs moved from the United States (high-wage) to Mexico (low-wage). The rise in within-country inequality in the United States is coupled with a rise in the inequality footprint in the past two decades (see Fig. S2). Fig. S2 shows a small decrease in inequality after 2003 with an increase following the financial crisis and a slight decrease again in the years during Barack Obama's presidency. This is perhaps because his election campaign included amending the NAFTA and its outsourcing of jobs to other countries.

Also in the United Kingdom within-country inequality is higher than its total inequality footprint. Our results show that the inequality footprint increased after the Tony Blair era with a decrease in 2003 and then increased again after the global financial crisis and recession in the United Kingdom after 2008 (see Fig. S2). The increasing inequality footprint after the recession may be due to rising commodity prices in the United Kingdom after 2008 [48]. Other researchers [54] attribute this rising inequality to a decline in the progressivity of the personal income tax schedule of the overall tax system in the United Kingdom and the United States and others [27] attribute the increase in within-country inequality in the United Kingdom and United States to the Reagan–Thatcher era which gave the green light to financial deregulation and dependence on foreign labour (labour embodied) through trading activities.

Russia occupies an unusual position at the bottom of the rank, because both its inequality footprint and its own Gini index are lower than those of similarly ranked countries such as Brazil and South Africa (see Table S6 in File S1). This is because approximately 62% of Russia's total share of embodied labour arrives from the Commonwealth of Independent States where inequality is low (Table 1). Most of its imported commodities are from agriculture or are raw materials (Table 1). Fig. S2 shows that the inequality footprint and within-country inequality increased in Russia after the collapse of the Soviet Union. The increase in the inequality footprint occurred because either within-Ukraine inequality increased in the 1990s (our findings showed that the highest labour embodied in the past two decades was from Ukraine (about 24% in 2010) [47] or Russia's other trading partners were from unequal countries. Also, the figure shows a decrease in the inequality footprint in 2000s, which is either because of the decreasing within-Ukraine inequality [47] in the 2000s or the economic crisis at the end of the 1990s in Russia.

Russia's low inequality footprint is reflected in its recent history. After the collapse of the former Soviet Union, tight economic links amongst the newly independent republics and the former Eastern Bloc continued [49]. Most of Russia's current trade partners achieved or maintained low inequality, either by keeping parts of the Soviet-style social systems such as pension benefits, and by avoiding economic reforms such as privatization [25], or, like Lithuania, Eastern Germany and Poland, by joining the European Union. On the other hand, Russia experienced a significant increase in within-country inequality due to the privatization of state-owned enterprises and inequality in wages within the private sector between men, women, and older workers [7]. However, because of the equality of its trading partners its inequality footprint remains low.

Investigating the main imported commodities between developing countries (Table 1) shows that, for example, China and Thailand import chiefly raw materials and intermediate goods. China imports raw materials such as natural rubber from Thailand and Malaysia, iron ore from Russia and Brazil, and wood in rough from Madagascar and intermediate products (electronic integrated circuits from the Philippines and Malaysia). Thailand also imports raw materials (or natural resources) from Russia (petroleum oil) and intermediate goods from Philippines and Malaysia (electronic integrated circuits). By recognizing the flows of embodied labour (or employment footprint) and the commodities traded between developing (or maybe developed) countries we provide information that can be used to support a policy agenda to address inequality within those countries (more details in conclusion).

The remaining countries with an inequality footprint higher than their own Gini include Brazil and South Africa, which are both characterised by high levels of inequality, probably because both lack policies to provide a comprehensive social security net, and both have experienced a rise in the wages gap between skilled and unskilled labour in rural and urban regions [30], [60]. In Brazil, the main agricultural products that may be implicated in within-country inequality are soya bean and fruit juices. Labour embodied in these commodities is exported to the Netherlands, Norway, and Austria, thus increasing the inequality footprints of those countries. Ores and wood pulp from Brazil also play a large part in increasing inequality footprints of importing countries (Table 1). In South Africa, inequality has historical roots going back to the beginning of the last century and apartheid when 10% of the population (i.e. white) held 50% of the total share of income [60] while the other racial groups (coloured 8%, Indian/Asian 2%, and African 80%) held 10%, 32%, and 8% respectively. During the last two decades inequality has declined between racial groups through the inclusion of blacks in the mining industry, which has allowed African workers to be classified as employees [29]. However inequality has risen within racial groups especially within African groups because of the large number of uneducated, unskilled workers and the small number of more highly paid educated workers [29]. Despite its geographical location surrounded by highly unequal countries, such as Namibia, Botswana and Madagascar, the inequality footprint of South Africa is equal to the footprint of some developed countries (Table 1). This is probably because embodied labour imported from countries that have a within-country Gini index above 0.4 is less than from countries that have a within-country Gini index below 0.4 (Table 1).

Our results show that the highest inequality footprint in the world is that of Namibia (0.5), because approximately 81% of total labour embodied in imports is from countries that have high levels of inequality (for example South Africa 65%, Russia 6%, and China 3%). However Fig. 3 does not show Namibia in the bottom-ranking countries because its own inequality is also the highest in the world (about 0.64 according to the World Bank). Therefore there is little difference between its within-country inequality and its inequality footprint.

The inequality footprint can add to our understanding of within-country inequality tracking implicated-commodities moving around the world. It also facilitates the distinguishing of problematic unequal labour from equal labour worldwide just as we already distinguish ‘scarce water’ from ‘non-scarce water’ [22] and ethical labour from unethical labour such as child labour [5]. Our work provides another tool that can assist businesses, government and non-government organizations to identify areas of responsibility and take action to make the world a better place.

## Conclusions

Both developing countries (producers) and developed countries (consumers) may suffer from inequality because of trade activities. Our findings show that the major commodities associated with wage inequality during production are agricultural and electronic commodities. Most developing countries who deal in these commodities have an inequality footprint less than their within-country Gini index. Among developed countries only the United States and the United Kingdom have within-country inequality higher than their inequality footprint. The United States shows an increasing inequality footprint during the Clinton era and especially after the NAFTA treaty came into force and then decreasing inequality footprints after 2003 and increasing again after the financial crisis of 2007 and slightly decreasing during Barack Obama's presidency. The United Kingdom shows that the inequality footprint increased after the Tony Blair era with a decrease in 2003 and then increasing again after the global financial crisis and recession in the United Kingdom after 2008. Both the United States and the United Kingdom showed a decrease in their inequality footprint after 2003 and this may be related to the war in Iraq and their relationships to Russia and China. Russia has demonstrated one of the lowest inequality footprints in the Commonwealth of Independent States and in the world.

We began this paper with a reference to the first 1990–2015 Millennium Development Goal; we end with a comment on the post-2015 agenda. The UN's Task Team on the post-2015 UN development agenda convened to address the issue of inequalities concludes that inequalities must be addressed along with human rights, peace, security and sustainability as a cornerstone of the society that we want to live in and pass on to the next generation [56].

Any new policy must be designed from a global perspective to reduce inequality. The UN Systems Task Team on the Post-2015 UN Development Agenda [56] recommends that there be a specific post-2015 goal on inequality with accompanying targets and equity-weighting indicators including income distribution. This they acknowledge will require investment in data collection, analysis and use, requiring a flexible standard measurement to capture, track and reflect inequalities and provide for transparency and accountability in progress. The Task Team further linked inequality to sustainability as a cornerstone of this agenda. It is difficult for individual countries to take action on what is a global problem. One way to address this, suggested by Basu [4], would be to create a global body to coordinate inter-country anti-poverty and anti-inequality policies, operating in the same way as the WTO helps mitigate problems in trade and the UNEP in environmental problems [4]. This is where the inequality footprint can play a role. It provides a map of the movement of embodied labour across countries and detailed information about which commodities are the most labour-intensive and may be implicated in within-country inequality.

The inequality footprint can add to our understanding of within-country inequality tracking implicated-commodities moving around the world. It also facilitates the distinguishing of problematic unequal labour from equal labour worldwide just as we already distinguish ‘scarce water’ from ‘non-scarce water’ [22] and ethical labour from unethical labour such as child labour [5]. Our work provides another tool that can assist businesses, government and non-government organizations to identify areas of responsibility and take action to make the world a better place.

## Supporting Information

Figure S1 Some examples of Lorenz curves calculated from income and employment quintiles or deciles to determine the Gini index of nations (circles represent the data [1], [2], [8], [9], [12] and the lines represent the power function approximation we used (these data populate *I* and *P* in section 3.4)).
(TIF)

Figure S2Example of time series of within-country Gini index (populate 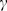 in section 3.4) against inequality footprint (populate 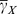 in section 3.5) spanning 1990–2010 for the top and bottom two countries of Fig. 3 and including United Kingdom and United States (each year is represented by a dot point and 2010 is represented by the arrowhead).(TIF)

Figure S3Example of time series of within-country Gini index (populate 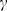 in section 3.4) against inequality footprint (populate 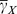 in section 3.5) spanning 1990–2010 for some of the Latin America countries (1990 is represented by the first point and 2010 is represented by the arrowhead).(TIF)

Figure S4Example of time series of within-country Gini index (populate 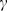 in section 3.4) against inequality footprint (populate 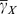 in section 3.5) spanning 1990–2010 for some of the European countries (1990 is represented by the first point and 2010 is represented by the arrowhead).(TIF)

File S1 Various supporting text and tables.
(DOCX)
