# Supplementary figures and images for: The Inequality Footprints of Nations: A Novel Approach to Quantitative Accounting of Income Inequality
Source: PLoS One. 2014 Oct 29;9(10):e110881. doi: 10.1371/journal.pone.0110881 (PMC4212986; doi:10.1371/journal.pone.0110881)

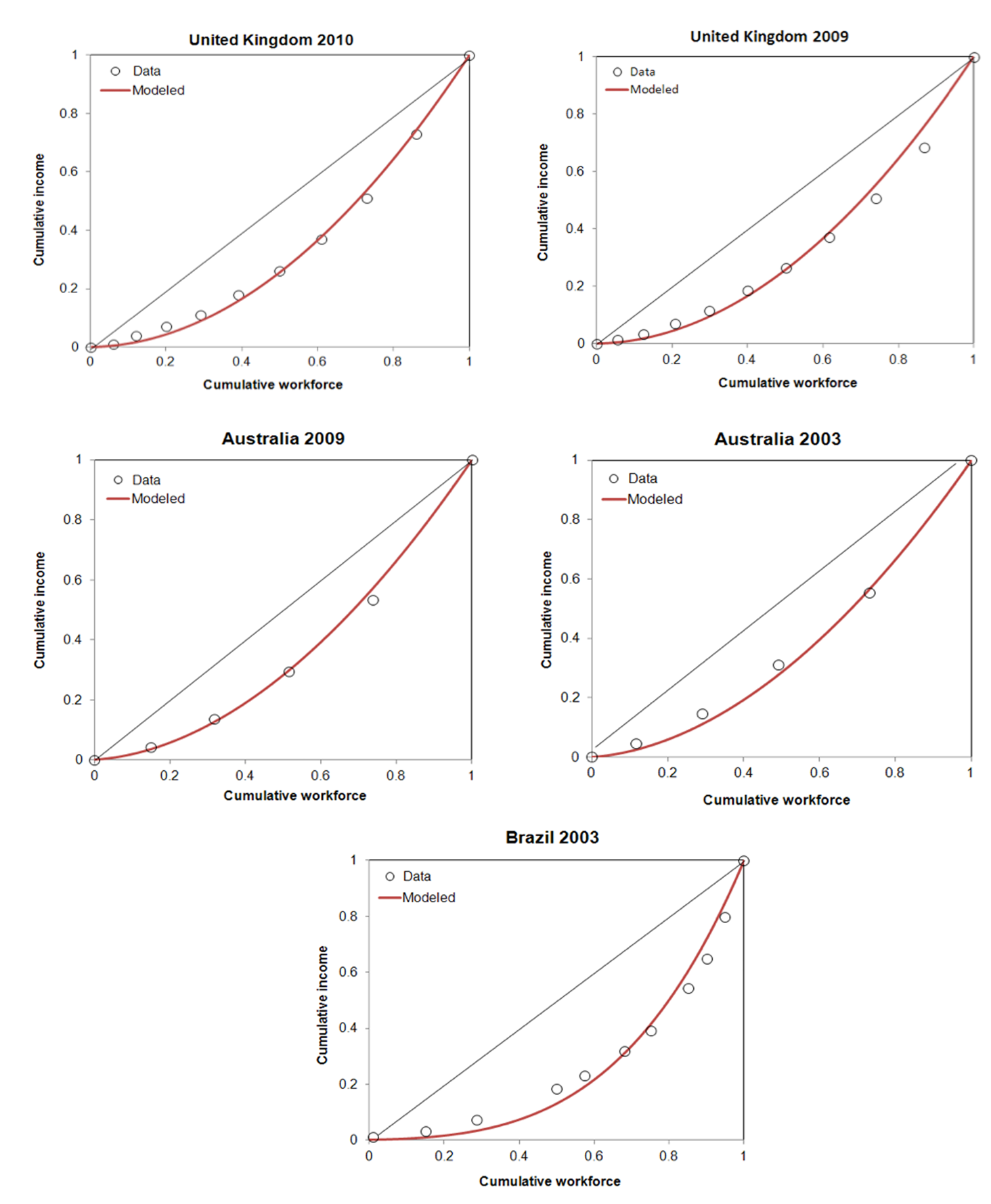

Supplement: Figure S1 — Some examples of Lorenz curves calculated from income and employment quintiles or deciles to determine the Gini index of nations (circles represent the data [1], [2], [8], [9], [12] and the lines represent the power function approximation we used (these data populate I and P in section 3.4)). (TIF) [file pone.0110881.s001.tif]

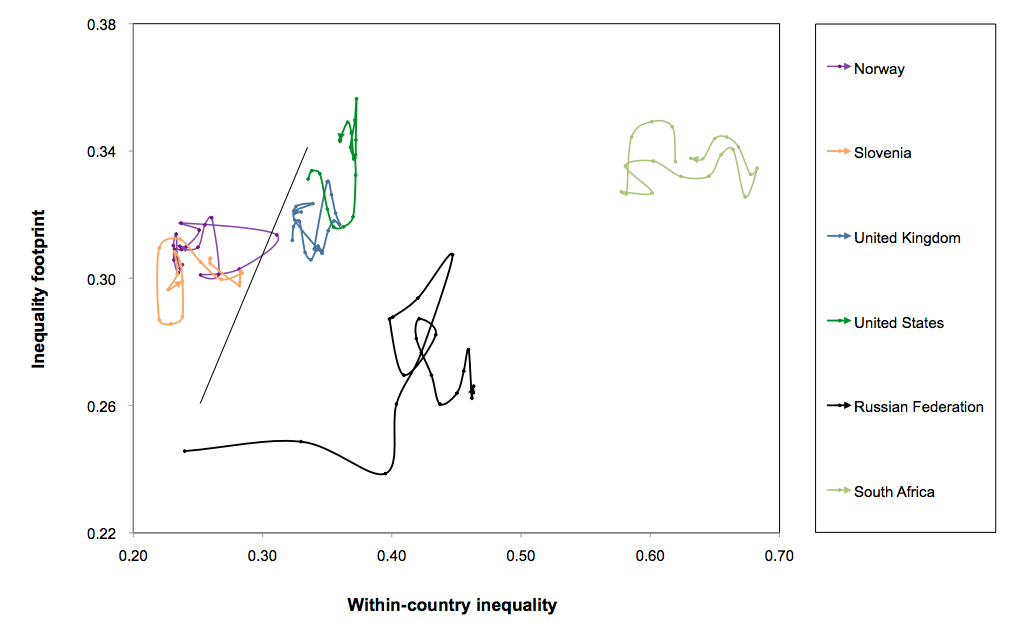

Supplement: Figure S2 — Example of time series of within-country Gini index (populate in section 3.4) against inequality footprint (populate in section 3.5) spanning 1990–2010 for the top and bottom two countries of Fig. 3 and including United Kingdom and United States (each year is represented by a dot point and 2010 is represented by the arrowhead). (TIF) [file pone.0110881.s002.tif]

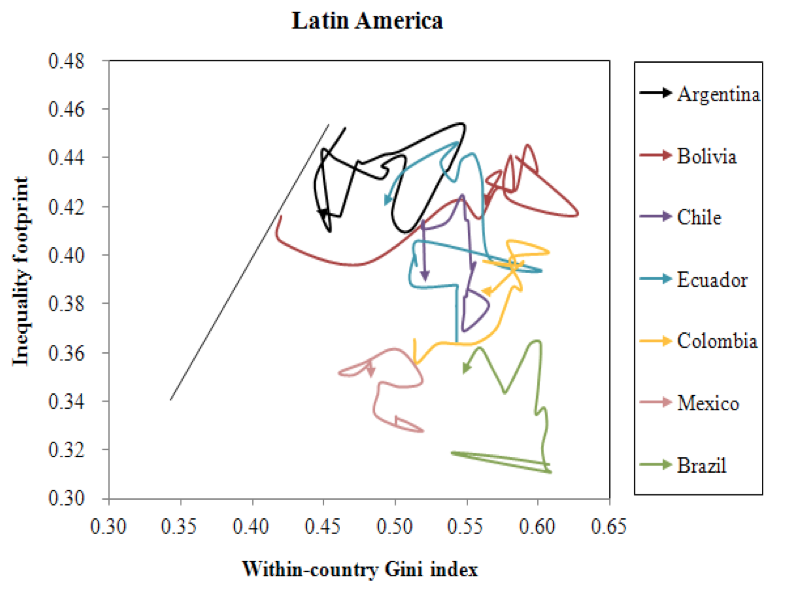

Supplement: Figure S3 — Example of time series of within-country Gini index (populate in section 3.4) against inequality footprint (populate in section 3.5) spanning 1990–2010 for some of the Latin America countries (1990 is represented by the first point and 2010 is represented by the arrowhead). (TIF) [file pone.0110881.s003.tif]

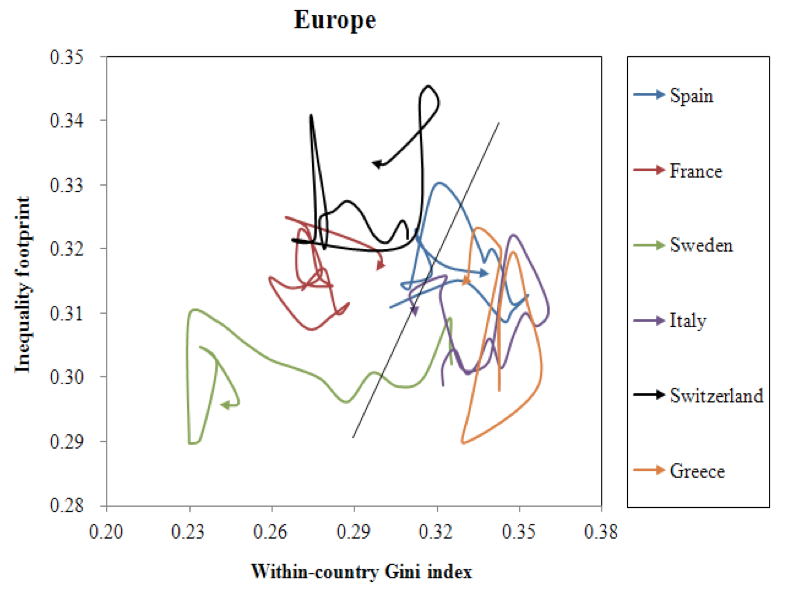

Supplement: Figure S4 — Example of time series of within-country Gini index (populate in section 3.4) against inequality footprint (populate in section 3.5) spanning 1990–2010 for some of the European countries (1990 is represented by the first point and 2010 is represented by the arrowhead). (TIF) [file pone.0110881.s004.tif]
