# Supplementary material for: The Inequality Footprints of Nations: A Novel Approach to Quantitative Accounting of Income Inequality
Source: PLoS One. 2014 Oct 29;9(10):e110881. doi: 10.1371/journal.pone.0110881 (PMC4212986; doi:10.1371/journal.pone.0110881)
Supplement: File S1 — Various supporting text and tables. (DOCX) [file pone.0110881.s005.docx]

***Appendix 1:* List of countries covered in this paper**

| UN code | | Name |  | Sectors (Number of industries/products) |
| --- | --- | --- | --- | --- |
|  |  |  |  |  |
| 4 |  | Afghanistan |  | 26/0 |
| 8 |  | Albania |  | 26/0 |
| 12 |  | Algeria |  | 26/0 |
| 20 |  | Andorra |  | 26/0 |
| 24 |  | Angola |  | 26/0 |
| 28 |  | Antigua and Barbuda |  | 26/0 |
| 32 |  | Argentina |  | 125/196 |
| 51 |  | Armenia |  | 26/0 |
| 533 |  | Aruba |  | 26/0 |
| 36 |  | Australia |  | 345/345 |
| 40 |  | Austria |  | 61/61 |
| 31 |  | Azerbaijan |  | 26/0 |
| 44 |  | Bahamas |  | 26/0 |
| 48 |  | Bahrain |  | 26/0 |
| 50 |  | Bangladesh |  | 26/0 |
| 52 |  | Barbados |  | 26/0 |
| 112 |  | Belarus |  | 26/0 |
| 56 |  | Belgium |  | 61/61 |
| 84 |  | Belize |  | 26/0 |
| 204 |  | Benin |  | 26/0 |
| 60 |  | Bermuda |  | 26/0 |
| 64 |  | Bhutan |  | 26/0 |
| 68 |  | Bolivia |  | 37/37 |
| 70 |  | Bosnia and Herzegovina |  | 26/0 |
| 72 |  | Botswana |  | 26/0 |
| 76 |  | Brazil |  | 56/111 |
| 92 |  | British Virgin Islands |  | 26/0 |
| 96 |  | Brunei Darussalam |  | 26/0 |
| 100 |  | Bulgaria |  | 26/0 |
| 854 |  | Burkina Faso |  | 26/0 |
| 108 |  | Burundi |  | 26/0 |
| 116 |  | Cambodia |  | 26/0 |
| 120 |  | Cameroon |  | 26/0 |
| 124 |  | Canada |  | 49/0 |
| 132 |  | Cape Verde |  | 26/0 |
| 136 |  | Cayman Islands |  | 26/0 |
| 140 |  | Central African Republic |  | 26/0 |
| 148 |  | Chad |  | 26/0 |
| 152 |  | Chile |  | 75/75 |
| 156 |  | China |  | 0/123 |
| 170 |  | Colombia |  | 60/60 |
| 178 |  | Congo |  | 26/0 |
| 188 |  | Costa Rica |  | 26/0 |
| 191 |  | Croatia |  | 26/0 |
| 192 |  | Cuba |  | 26/0 |
| 196 |  | Cyprus |  | 26/0 |
| 203 |  | Czech Republic |  | 61/61 |
| 384 |  | Côte d'Ivoire |  | 26/0 |
| 408 |  | Democratic People's Republic of Korea |  | 26/0 |
| 180 |  | Democratic Republic of the Congo, previously Zaïre | | 26/0 |
| 208 |  | Denmark |  | 131/0 |
| 262 |  | Djibouti |  | 26/0 |
| 214 |  | Dominican Republic |  | 26/0 |
| 218 |  | Ecuador |  | 49/61 |
| 818 |  | Egypt |  | 26/0 |
| 222 |  | El Salvador |  | 26/0 |
| 232 |  | Eritrea |  | 26/0 |
| 233 |  | Estonia |  | 61/61 |
| 231 |  | Ethiopia |  | 26/0 |
| 242 |  | Fiji |  | 26/0 |
| 246 |  | Finland |  | 61/61 |
| 250 |  | France |  | 61/61 |
| 258 |  | French Polynesia |  | 26/0 |
| 266 |  | Gabon |  | 26/0 |
| 270 |  | Gambia |  | 26/0 |
| 268 |  | Georgia |  | 47/68 |
| 276 |  | Germany |  | 0/72 |
| 288 |  | Ghana |  | 26/0 |
| 300 |  | Greece |  | 61/61 |
| 304 |  | Greenland |  | 31/0 |
| 320 |  | Guatemala |  | 26/0 |
| 324 |  | Guinea |  | 26/0 |
| 328 |  | Guyana |  | 26/0 |
| 332 |  | Haiti |  | 26/0 |
| 340 |  | Honduras |  | 26/0 |
| 344 |  | Hong Kong |  | 38/38 |
| 348 |  | Hungary |  | 61/61 |
| 352 |  | Iceland |  | 26/0 |
| 356 |  | India |  | 116/116 |
| 360 |  | Indonesia |  | 0/77 |
| 364 |  | Iran |  | 100/148 |
| 368 |  | Iraq |  | 26/0 |
| 372 |  | Ireland |  | 61/61 |
| 376 |  | Israel |  | 163/163 |
| 380 |  | Italy |  | 61/61 |
| 388 |  | Jamaica |  | 26/0 |
| 392 |  | Japan |  | 0/402 |
| 400 |  | Jordan |  | 26/0 |
| 398 |  | Kazakhstan |  | 0/121 |
| 404 |  | Kenya |  | 51/51 |
| 414 |  | Kuwait |  | 55/0 |
| 417 |  | Kyrgyzstan |  | 89/87 |
| 418 |  | Lao People's Democratic Republic |  | 26/0 |
| 428 |  | Latvia |  | 61/61 |
| 422 |  | Lebanon |  | 26/0 |
| 426 |  | Lesotho |  | 26/0 |
| 430 |  | Liberia |  | 26/0 |
| 434 |  | Libyan Arab Jamahiriya |  | 26/0 |
| 438 |  | Liechtenstein |  | 26/0 |
| 440 |  | Lithuania |  | 61/61 |
| 442 |  | Luxembourg |  | 26/0 |
| 446 |  | Macao Special Administrative Region of China |  | 26/0 |
| 450 |  | Madagascar |  | 26/0 |
| 454 |  | Malawi |  | 26/0 |
| 458 |  | Malaysia |  | 0/98 |
| 462 |  | Maldives |  | 26/0 |
| 466 |  | Mali |  | 26/0 |
| 470 |  | Malta |  | 61/61 |
| 478 |  | Mauritania |  | 26/0 |
| 480 |  | Mauritius |  | 57/67 |
| 484 |  | Mexico |  | 80/80 |
| 492 |  | Monaco |  | 26/0 |
| 496 |  | Mongolia |  | 26/0 |
| 499 |  | Montenegro |  | 26/0 |
| 504 |  | Morocco |  | 26/0 |
| 508 |  | Mozambique |  | 26/0 |
| 104 |  | Myanmar |  | 26/0 |
| 516 |  | Namibia |  | 26/0 |
| 524 |  | Nepal |  | 26/0 |
| 528 |  | Netherlands |  | 61/61 |
| 530 |  | Netherlands Antilles |  | 16/41 |
| 540 |  | New Caledonia |  | 26/0 |
| 554 |  | New Zealand |  | 127/210 |
| 558 |  | Nicaragua |  | 26/0 |
| 562 |  | Niger |  | 26/0 |
| 566 |  | Nigeria |  | 26/0 |
| 578 |  | Norway |  | 61/61 |
| 275 |  | West Bank and Gaza Strip |  | 26/0 |
| 512 |  | Oman |  | 26/0 |
| 586 |  | Pakistan |  | 26/0 |
| 591 |  | Panama |  | 26/0 |
| 598 |  | Papua New Guinea |  | 26/0 |
| 600 |  | Paraguay |  | 34/47 |
| 604 |  | Peru |  | 46/46 |
| 608 |  | Philippines |  | 0/77 |
| 616 |  | Poland |  | 61/61 |
| 620 |  | Portugal |  | 61/61 |
| 634 |  | Qatar |  | 26/0 |
| 410 |  | Republic of Korea |  | 0/78 |
| 498 |  | Republic of Moldova |  | 26/0 |
| 642 |  | Romania |  | 61/61 |
| 643 |  | Russian Federation |  | 49/0 |
| 646 |  | Rwanda |  | 26/0 |
| 882 |  | Samoa |  | 26/0 |
| 674 |  | San Marino |  | 26/0 |
| 678 |  | Sao Tome and Principe |  | 26/0 |
| 682 |  | Saudi Arabia |  | 26/0 |
| 686 |  | Senegal |  | 26/0 |
| 688 |  | Serbia |  | 26/0 |
| 690 |  | Seychelles |  | 26/0 |
| 694 |  | Sierra Leone |  | 26/0 |
| 702 |  | Singapore |  | 154/154 |
| 703 |  | Slovakia |  | 61/61 |
| 705 |  | Slovenia |  | 61/61 |
| 706 |  | Somalia |  | 26/0 |
| 710 |  | South Africa |  | 95/96 |
| 724 |  | Spain |  | 76/119 |
| 144 |  | Sri Lanka |  | 26/0 |
| 736 |  | Sudan |  | 26/0 |
| 740 |  | Suriname |  | 26/0 |
| 748 |  | Swaziland |  | 26/0 |
| 752 |  | Sweden |  | 61/61 |
| 756 |  | Switzerland |  | 43/43 |
| 760 |  | Syrian Arab Republic |  | 26/0 |
| 761 |  | Taiwan |  | 0/163 |
| 762 |  | Tajikistan |  | 26/0 |
| 764 |  | Thailand |  | 0/180 |
| 807 |  | Macedonia |  | 61/61 |
| 768 |  | Togo |  | 26/0 |
| 780 |  | Trinidad and Tobago |  | 26/0 |
| 788 |  | Tunisia |  | 26/0 |
| 792 |  | Turkey |  | 61/61 |
| 795 |  | Turkmenistan |  | 26/0 |
| 800 |  | Uganda |  | 26/0 |
| 804 |  | Ukraine |  | 0/121 |
| 784 |  | United Arab Emirates |  | 26/0 |
| 826 |  | United Kingdom |  | 511/511 |
| 834 |  | Tanzania |  | 26/0 |
| 840 |  | United States |  | 429/429 |
| 858 |  | Uruguay |  | 84/103 |
| 860 |  | Uzbekistan |  | 0/123 |
| 548 |  | Vanuatu |  | 26/0 |
| 862 |  | Venezuela |  | 122/122 |
| 704 |  | Viet Nam |  | 0/113 |
| 887 |  | Yemen |  | 26/0 |
| 894 |  | Zambia |  | 26/0 |
| 716 |  | Zimbabwe |  | 26/0 |

***Appendix 2:* Countries and years for which data are available;**

X = data available

Data sources: (SWIID version 3.1 [20]), OCED database, and the World Bank database.

| **Country** | **1990** | **1991** | **1992** | **1993** | **1994** | **1995** | **1996** | **1997** | **1998** | **1999** | **2000** | **2001** | **2002** | **2003** | **2004** | **2005** | **2006** | **2007** | **2008** | **2009** | **2010** |
| --- | --- | --- | --- | --- | --- | --- | --- | --- | --- | --- | --- | --- | --- | --- | --- | --- | --- | --- | --- | --- | --- |
| Afghanistan | 0 |  |  |  |  |  |  |  |  |  |  |  |  |  |  |  |  |  | x |  |  |
| Albania | 0 |  |  |  |  |  | x | x | x | x | x | x | x | x | x | x |  |  | x |  |  |
| Algeria | x | x | x | x | x | x | x | x | x | x | x | x | x | x | x | x |  |  |  |  |  |
| Andorra | 0 |  |  |  |  |  |  |  |  |  |  | x | x | x |  |  |  |  | x |  |  |
| Angola | 0 |  |  |  |  | x | x | x | x | x | x | x | x | x | x | x |  |  |  |  | x |
| Antigua and Barbuda | 0 |  |  |  |  |  |  |  |  |  |  | x | x |  |  |  |  |  |  |  |  |
| Argentina | x | x | x | x | x | x | x | x | x | x | x | x | x | x | x | x | x | x | x | x | x |
| Armenia | x | x | x | x | x | x | x | x | x | x | x | x | x | x | x | x | x | x |  |  |  |
| Aruba | 0 |  |  |  |  |  |  |  |  |  |  |  |  |  |  |  | x |  |  |  |  |
| Australia | x | x | x | x | x | x | x | x | x | x | x | x | x | x | x | x | x | x | x | x | x |
| Austria | x | x | x | x | x | x | x | x | x | x | x | x | x | x | x | x | x | x | x | x | x |
| Azerbaijan | x | x | x | x | x | x | x | x | x | x | x | x | x | x | x | x | x | x | x |  |  |
| Bahamas | x | x | x | x | x | x | x | x | x | x | x | x | x | x | x |  |  |  |  |  |  |
| Bahrain | 0 |  |  |  |  |  |  |  |  |  |  |  |  |  | x |  |  |  |  |  |  |
| Bangladesh | x | x | x | x | x | x | x | x | x | x | x | x | x | x | x | x | x | x | x | x | x |
| Barbados | x | x | x | x | x | x | x | x |  |  |  |  |  |  |  |  |  |  |  |  |  |
| Belarus | x | x | x | x | x | x | x | x | x | x | x | x | x | x | x | x | x | x | x |  |  |
| Belgium | x | x | x | x | x | x | x | x | x | x | x | x | x | x | x | x | x | x | x | x | x |
| Belize | 0 |  |  | x | x | x | x | x | x | x |  |  |  |  |  | x | x |  |  |  |  |
| Benin | 0 |  |  |  |  |  |  |  |  |  |  |  |  | x | x | x | x |  |  |  |  |
| Bermuda | 0 |  |  |  |  |  |  |  |  |  |  |  |  |  |  |  | x |  |  |  |  |
| Bhutan | 0 |  |  |  |  |  |  |  |  |  |  |  |  | x | x | x | x | x |  |  |  |
| Bolivia | x | x | x | x | x | x | x | x | x | x | x | x | x | x | x | x | x | x |  |  |  |
| Bosnia and Herzegovina | 0 | x | x | x | x | x | x | x | x | x | x | x | x | x | x | x |  | x |  | x |  |
| Botswana | x | x | x | x | x | x | x | x | x | x | x | x | x | x | x | x |  |  |  | x | x |
| Brazil | x | x | x | x | x | x | x | x | x | x | x | x | x | x | x | x | x | x | x | x | x |
| British Virgin Islands | 0 |  |  |  |  |  |  |  |  |  |  |  |  |  |  | x |  |  |  |  |  |
| Brunei Darussalam | 0 |  |  |  |  |  |  |  |  |  |  | x |  |  |  |  |  |  |  |  |  |
| Bulgaria | x | x | x | x | x | x | x | x | x | x | x | x | x | x | x | x | x | x | x | x | x |
| Burkina Faso | 0 |  |  |  | x | x | x | x | x | x | x | x | x | x |  |  |  |  |  | x |  |
| Burundi | 0 |  | x | x | x | x | x | x | x | x | x | x | x | x | x | x | x |  |  |  |  |
| Cambodia | 0 |  |  |  | x | x | x | x | x | x | x | x | x | x | x |  |  | x | x |  | x |
| Cameroon | x | x | x | x | x | x | x | x | x | x | x | x | x |  |  |  |  | x |  |  |  |
| Canada | x | x | x | x | x | x | x | x | x | x | x | x | x | x | x | x | x | x | x | x |  |
| Cape Verde | x | x | x | x | x | x | x | x | x | x | x | x | x | x | x | x |  |  |  |  |  |
| Cayman Islands | 0 |  | x |  |  |  |  |  |  |  |  |  |  | x |  |  |  |  | x |  |  |
| Central African Republic | o |  | x | x | x | x | x | x | x | x | x | x | x | x |  |  |  |  | x |  |  |
| Chad | |  |  |  |  |  |  |  |  |  |  |  | x | x | x | x |  |  |  |  |  |
| Chile | x | x | x | x | x | x | x | x | x | x | x | x | x | x | x | x | x | x | x | x |  |
| China | x | x | x | x | x | x | x | x | x | x | x | x | x | x | x | x | x |  |  | x | x |
| Colombia | x | x | x | x | x | x | x | x | x | x | x | x | x | x | x | x | x | x | x | x |  |
| Congo | 0 |  |  |  |  |  |  |  |  |  |  |  |  |  |  | x | x |  |  |  |  |
| Costa Rica | x | x | x | x | x | x | x | x | x | x | x | x | x | x | x | x | x | x | x | x |  |
| Croatia | x | x | x | x | x | x | x | x | x | x | x | x | x | x | x | x | x | x | x | x |  |
| Cuba | 0 |  |  |  |  |  |  |  |  |  |  |  | x |  |  |  |  |  |  |  |  |
| Cyprus | x | x | x | x | x | x | x | x | x | x | x | x | x | x | x | x | x | x | x | x |  |
| Czech Republic | x | x | x | x | x | x | x | x | x | x | x | x | x | x | x | x | x | x | x | x | x |
| côte d'ivoire | x | x | x | x | x | x | x | x | x | x | x | x | x |  |  |  |  |  |  |  |  |
| North Korea | x |  |  |  |  |  | x |  | x |  |  |  |  |  |  | x |  |  |  |  | x |
| Democratic Republic of the Congo, previously Zaïre | 0 |  |  |  |  |  |  |  |  |  |  |  |  |  | x |  |  |  |  |  |  |
| Denmark | x | x | x | x | x | x | x | x | x | x | x | x | x | x | x | x | x | x | x | x | x |
| Djibouti | | |  |  |  | x | x | x | x | x | x | x | x | x | x | x |  |  |  |  |  |
| Dominican Republic | x | x | x | x | x | x | x | x | x | x | x | x | x | x | x | x | x | x | x | x | x |
| Ecuador | x | x | x | x | x | x | x | x | x | x | x | x | x | x | x | x | x | x | x | x |  |
| Egypt | x | x | x | x | x | x | x | x | x | x | x | x | x | x | x | x | x | x | x |  |  |
| El Salvador | x | x | x | x | x | x | x | x | x | x | x | x | x | x | x | x | x | x | x |  |  |
| Eritrea | 0 |  |  |  |  |  |  |  |  |  |  |  |  |  |  |  |  |  | x |  |  |
| Estonia | x | x | x | x | x | x | x | x | x | x | x | x | x | x | x | x | x | x | x | x | x |
| Ethiopia | x | x | x | x | x | x | x | x | x | x | x | x | x | x | x | x |  |  |  |  |  |
| Fiji | x | x | x |  |  |  |  |  | x |  | x |  |  | x |  |  |  |  |  | x |  |
| Finland | x | x | x | x | x | x | x | x | x | x | x | x | x | x | x | x | x | x | x | x | x |
| France | x | x | x | x | x | x | x | x | x | x | x | x | x | x | x | x | x | x | x | x | x |
| French Polynesia | 0 |  |  |  |  |  |  |  |  |  |  |  | x |  |  |  |  |  |  |  |  |
| Gabon | x | x | x |  |  |  |  |  |  |  |  |  |  |  |  | x |  |  |  |  |  |
| Gambia | x | x | x | x | x | x | x | x | x | x | x | x | x | x |  |  |  |  |  |  |  |
| Georgia | x | x | x | x | x | x | x | x | x | x | x | x | x | x | x | x | x |  |  |  |  |
| Germany | x | x | x | x | x | x | x | x | x | x | x | x | x | x | x | x | x | x | x | x | x |
| Ghana | x | x | x | x | x | x | x | x | x | x | x | x | x | x | x | x | x |  |  |  |  |
| Greece | x | x | x | x | x | x | x | x | x | x | x | x | x | x | x | x | x | x | x | x | x |
| Greenland | 0 |  |  |  | x |  |  |  |  |  |  |  |  |  |  | x |  |  |  |  |  |
| Guatemala | x | x | x | x | x | x | x | x | x | x | x | x | x | x | x | x | x |  |  |  |  |
| Guinea | x | x | x | x | x | x | x | x | x | x | x | x | x | x | x | x | x |  |  |  |  |
| Guyana | | | x | x | x | x | x | x | x | x |  |  |  |  | x |  |  |  |  |  |  |
| Haiti | x | x | x | x | x | x | x | x | x | x | x | x |  |  |  |  | x | x | x |  |  |
| Honduras | x | x | x | x | x | x | x | x | x | x | x | x | x | x | x | x | x | x | x | x |  |
| Hong Kong | x | x | x | x | x | x | x | x | x | x | x | x | x | x | x | x | x |  | x |  |  |
| Hungary | x | x | x | x | x | x | x | x | x | x | x | x | x | x | x | x | x | x | x | x | x |
| Iceland | 0 | x | x | x | x | x | x | x | x | x | x | x | x | x | x | x | x | x | x | x | x |
| India | x | x | x | x | x | x | x | x | x | x | x | x | x | x | x | x |  |  |  |  |  |
| Indonesia | x | x | x | x | x | x | x | x | x | x | x | x | x | x | x | x | x | x | x | x | x |
| Iran | x | x | x | x | x | x | x | x | x | x | x | x | x | x | x |  |  |  |  |  |  |
| Iraq | |  |  |  |  |  |  |  |  |  |  |  |  | x | x |  |  | x |  |  |  |
| Ireland | x | x | x | x | x | x | x | x | x | x | x | x | x | x | x | x | x | x | x | x |  |
| Israel | x | x | x | x | x | x | x | x | x | x | x | x | x | x | x | x |  |  |  | x | x |
| Italy | x | x | x | x | x | x | x | x | x | x | x | x | x | x | x | x | x | x | x | x | x |
| Jamaica | x | x | x | x | x | x | x | x | x | x | x | x | x | x | x |  |  |  |  |  |  |
| Japan | x | x | x | x | x | x | x | x | x | x | x | x | x | x | x | x | x | x | x | x | x |
| Jordan | x | x | x | x | x | x | x | x | x | x | x | x | x | x | x | x | x |  | x |  |  |
| Kazakhstan | x | x | x | x | x | x | x | x | x | x | x | x | x | x | x | x | x | x | x | x | x |
| Kenya | x | x | x | x | x | x | x | x | x | x | x | x | x | x | x | x |  |  |  |  |  |
| Kuwait | 0 |  |  |  |  |  |  |  |  |  |  |  |  |  |  | x |  |  |  |  |  |
| Kyrgyzstan | x | x | x | x | x | x | x | x | x | x | x | x | x | x | x | x | x | x | x | x | x |
| Lao People's Democratic Republic | 0 |  | x | x | x | x | x | x | x | x | x | x | x | x | x | x | x | x |  |  |  |
| Latvia | x | x | x | x | x | x | x | x | x | x | x | x | x | x | x | x | x | x | x | x | x |
| Lebanon | | |  |  |  |  |  | x | x | x | x | x | x | x | x | x |  |  |  | x |  |
| Lesotho | x | x | x | x | x | x | x | x | x | x | x | x | x | x | x | x |  |  |  |  |  |
| Liberia | x |  |  |  |  |  |  |  |  |  |  |  |  |  |  | x | x | x |  |  |  |
| Libyan Arab Jamahiriya | 0 |  |  |  |  |  |  |  |  |  |  | x |  |  |  |  |  |  |  |  |  |
| Liechtenstein | 0 |  |  |  |  |  |  |  |  |  |  |  | x |  |  |  |  |  |  |  |  |
| Lithuania | x | x | x | x | x | x | x | x | x | x | x | x | x | x | x | x | x | x | x | x | x |
| Luxembourg | x | x | x | x | x | x | x | x | x | x | x | x | x | x | x | x | x | x | x | x | x |
| Macau | x | x | x | x | x | x | x | x | x | x | x | x | x | x | x | x | x | x |  |  |  |
| Madagascar | x | x | x | x | x | x | x | x | x | x | x | x | x | x | x | x |  |  |  |  |  |
| Malawi | x | x | x | x | x | x | x | x | x | x | x | x | x | x | x | x |  |  |  |  |  |
| Malaysia | x | x | x | x | x | x | x | x | x | x | x | x | x | x | x | x |  | x |  | x |  |
| Maldives | 0 |  |  |  |  |  |  |  | x |  |  |  |  | x | x |  |  |  |  |  |  |
| Mali | x | x | x | x | x | x | x | x | x | x | x | x | x | x | x | x | x |  |  |  |  |
| Malta | 0 |  |  |  |  |  |  |  |  |  | x | x | x | x | x | x | x | x | x | x | x |
| Mauritania | x | x | x | x | x | x | x | x | x | x | x |  |  |  | x |  |  |  | x |  |  |
| Mauritius | x | x | x | x | x | x | x | x | x | x | x | x | x | x | x | x | x |  |  |  |  |
| Mexico | x | x | x | x | x | x | x | x | x | x | x | x | x | x | x | x | x | x | x | x | x |
| Monaco | 0 |  | x |  |  |  |  | x | x | x |  | x | x | x | x | x | x | x | x | x | x |
| Mongolia | 0 |  |  |  |  | x | x | x | x | x | x | x | x | x | x | x | x |  |  |  |  |
| Montenegro | 0 |  |  |  |  |  |  |  |  |  | x | x | x | x | x | x | x | x | x |  |  |
| Morocco | x | x | x | x | x | x | x | x | x | x | x | x | x | x | x | x | x | x |  |  |  |
| Mozambique | 0 |  |  |  |  |  | x | x | x | x | x | x | x | x | x | x |  |  |  |  |  |
| Myanmar | 0 |  |  |  |  |  |  |  |  |  |  |  |  |  | x |  |  |  |  |  |  |
| Namibia | | |  | x | x | x | x | x | x | x | x | x | x | x | x | x |  |  | x |  |  |
| Nepal | x | x | x | x | x | x | x | x | x | x | x | x | x | x | x |  |  |  |  |  |  |
| Netherlands | x | x | x | x | x | x | x | x | x | x | x | x | x | x | x | x | x | x | x | x | x |
| Netherlands Antilles | 0 |  |  |  |  |  |  |  |  |  |  | x |  |  |  |  |  |  |  |  |  |
| New Caledonia | 0 |  |  |  |  |  |  |  |  |  |  |  |  |  |  | x |  |  |  |  |  |
| New Zealand | x | x | x | x | x | x | x | x | x | x | x | x | x | x | x | x | x | x |  |  |  |
| Nicaragua | x |  |  | x |  |  |  |  | x |  |  | x |  |  |  | x |  |  |  |  |  |
| Niger | | x | x | x | x | x | x | x | x | x | x | x | x | x | x | x |  |  |  |  |  |
| Nigeria | x | x | x | x | x | x | x | x | x | x | x | x | x | x | x |  |  |  |  |  |  |
| Norway | x | x | x | x | x | x | x | x | x | x | x | x | x | x | x | x | x | x | x | x | x |
| West Bank and Gaza Strip | 0 |  |  |  |  |  |  |  |  |  |  |  |  |  |  |  |  | x |  | x |  |
| Oman | 0 |  |  |  |  |  |  |  |  |  |  | x |  |  |  |  |  |  |  |  |  |
| Pakistan | x | x | x | x | x | x | x | x | x | x | x | x | x | x | x | x | x | x |  |  |  |
| Panama | x | x | x | x | x | x | x | x | x | x | x | x | x | x | x | x | x | x | x | x |  |
| Papua New Guinea | 0 |  |  |  |  | x | x | x | x | x | x | x | x | x | x | x |  |  |  |  |  |
| Paraguay | x | x | x | x | x | x | x | x | x | x | x | x | x | x | x | x | x | x | x | x |  |
| Peru | x | x | x | x | x | x | x | x | x | x | x | x | x | x | x | x | x | x | x | x |  |
| Philippines | x | x | x | x | x | x | x | x | x | x | x | x | x | x | x | x | x | x | x | x |  |
| Poland | x | x | x | x | x | x | x | x | x | x | x | x | x | x | x | x | x | x | x | x | x |
| Portugal | x | x | x | x | x | x | x | x | x | x | x | x | x | x | x | x | x | x | x | x | x |
| Qatar | 0 |  |  |  |  |  |  |  |  |  |  |  |  |  |  |  |  | x |  |  |  |
| Republic of Korea | x | x | x | x | x | x | x | x | x | x | x | x | x | x | x | x | x | x | x | x | x |
| Moldova | x | x | x | x | x | x | x | x | x | x | x | x | x | x | x | x | x | x | x | x | x |
| Romania | x | x | x | x | x | x | x | x | x | x | x | x | x | x | x | x | x | x | x | x | x |
| Russian Federation | x | x | x | x | x | x | x | x | x | x | x | x | x | x | x | x | x | x | x | x |  |
| Rwanda | 0 |  |  |  |  | x | x | x | x | x | x | x | x | x | x | x | x |  |  |  |  |
| Samoa | 0 |  |  |  |  |  |  |  | x |  |  |  |  |  |  |  |  |  |  |  |  |
| San Marino | 0 |  |  |  |  |  |  |  |  |  |  |  |  |  | x |  |  |  |  |  |  |
| Sao Tome and Principe | 0 |  |  |  |  |  |  |  |  |  |  | x |  |  |  |  |  |  |  |  |  |
| Saudi Arabia | 0 |  |  |  |  |  |  |  |  |  |  |  |  | x |  |  |  |  |  |  |  |
| Senegal | 0 | x | x | x | x | x | x | x | x | x | x | x | x | x | x | x |  |  |  |  |  |
| Serbia | | |  |  |  |  |  |  |  |  |  | x | x | x | x | x | x | x |  |  |  |
| Seychelles | 0 |  |  |  |  |  |  |  |  |  | x |  |  |  |  |  |  | x | x |  |  |
| Sierra Leone | x | x | x | x | x | x | x | x | x | x | x | x | x | x | x | x |  |  |  |  |  |
| Singapore | x | x | x | x | x | x | x | x | x | x | x | x | x | x | x | x | x | x | x | x |  |
| Slovakia | x | x | x | x | x | x | x | x | x | x | x | x | x | x | x | x | x | x | x | x | x |
| Slovenia | x | x | x | x | x | x | x | x | x | x | x | x | x | x | x | x | x | x | x | x | x |
| Somalia | 0 |  |  |  |  |  |  |  |  |  |  |  | x |  |  |  |  |  |  |  |  |
| South Africa | x | x | x | x | x | x | x | x | x | x | x | x | x | x | x | x | x | x | x | x |  |
| Spain | x | x | x | x | x | x | x | x | x | x | x | x | x | x | x | x | x | x | x | x | x |
| Sri Lanka | x | x | x | x | x | x | x | x | x | x | x | x | x |  |  |  |  | x |  |  |  |
| Sudan | 0 |  |  |  |  |  |  |  |  |  |  | x |  |  |  |  |  |  |  |  |  |
| Suriname | 0 |  |  |  |  |  |  |  |  | x | x | x | x | x | x | x |  |  |  |  |  |
| Swaziland | x |  |  |  | x | x | x | x | x | x | x | x | x | x | x | x |  |  |  | x | x |
| Sweden | x | x | x | x | x | x | x | x | x | x | x | x | x | x | x | x | x | x | x | x | x |
| Switzerland | x | x | x | x | x | x | x | x | x | x | x | x | x | x | x | x | x | x | x | x |  |
| Syrian Arab Republic | 0 |  |  |  |  |  |  |  |  |  |  |  |  | x |  |  |  |  |  |  |  |
| Taiwan | x | x | x | x | x | x | x | x | x | x | x | x | x | x | x | x |  |  |  |  |  |
| Tajikistan | x | x | x | x | x | x | x | x | x | x | x | x | x | x | x |  |  |  |  |  |  |
| Thailand | x | x | x | x | x | x | x | x | x | x | x | x | x | x | x |  | x |  | x | x |  |
| Macedonia | 0 |  |  |  |  |  |  |  | x |  | x |  | x | x | x | x | x |  | x | x | x |
| Togo | |  |  |  |  |  |  |  |  |  |  |  |  |  |  | x | x |  |  |  |  |
| Trinidad and Tobago | x | x | x | x | x | x | x | x | x | x | x | x | x | x | x | x |  |  | x | x | x |
| Tunisia | x | x | x | x | x | x | x | x | x | x | x | x | x | x | x | x |  |  |  |  |  |
| Turkey | x | x | x | x | x | x | x | x | x | x | x | x | x | x | x | x | x | x | x | x |  |
| Turkmenistan | x | x | x | x | x | x | x | x | x | x | x | x | x | x | x | x |  |  |  |  |  |
| Uganda | x | x | x | x | x | x | x | x | x | x | x | x | x | x | x | x | x |  |  |  |  |
| Ukraine | x | x | x | x | x | x | x | x | x | x | x | x | x | x | x | x | x | x |  |  |  |
| United Arab Emirates | 0 |  |  |  |  |  |  |  |  |  |  |  |  |  |  | x |  |  |  |  |  |
| United Kingdom | x | x | x | x | x | x | x | x | x | x | x | x | x | x | x | x | x | x | x | x | x |
| Tanzania | x | x | x | x | x | x | x | x | x | x | x | x |  |  |  |  |  | x |  |  |  |
| United States | x | x | x | x | x | x | x | x | x | x | x | x | x | x | x | x | x | x | x | x | x |
| Uruguay | x | x | x | x | x | x | x | x | x | x | x | x | x | x | x | x | x | x | x | x |  |
| Uzbekistan | x | x | x | x | x | x | x | x | x | x | x | x | x | x | x | x |  |  |  |  |  |
| Vanuatu | 0 |  |  |  | x |  |  |  |  |  |  |  |  |  |  |  |  |  | x |  |  |
| Venezuela | x | x | x | x | x | x | x | x | x | x | x | x | x | x | x | x | x | x | x | x | x |
| Viet Nam | x | x | x | x | x | x | x | x | x | x | x | x | x | x | x | x | x |  |  |  |  |
| Yemen | | | x | x | x | x | x | x | x | x | x | x | x | x | x | x |  |  |  |  |  |
| Zambia | x | x | x | x | x | x | x | x | x | x | x | x | x | x | x | x |  |  |  |  |  |
| Zimbabwe | x | x | x | x | x | x |  |  |  |  |  |  |  |  |  |  |  |  |  |  |  |

***Appendix 3:***

**3.1 What is a satellite account?**

Satellite accounts are used to expand the analytical capacity of national economic accounts in social and environmental areas [21]. Moreover, they link physical data sources to the monetary national account [22]. Satellite accounts are expressed in terms of satellite indicators.

Satellite indicators represent social and environmental impacts. For example, employment is an indicator of a particular social impact. Most satellite indicators can be broken down into sub-indicators [4] (Table S1 & S2).

**Table S31:** Example of social indicators

| Indicators | Sub-indicators |
| --- | --- |
| Job/Employment generation | Male |
|  | Female |
|  | Child labour |
| Family income | Male |
|  | Female |
|  | Age group |

**Table S32:** Example of environmental indicators

| Indicators | Sub-indicators |
| --- | --- |
| Greenhouse gas emissions | CO2 |
|  | CH4 |
|  | N2O |
|  | HFC |
| Energy use | Black coal |
|  | Electricity |
|  | Natural gas |
|  | Auto gasoline, leaded |
| Water use | Mains water |
|  | Self-supplied water |
|  | Reuse water |
|  | In-stream water |

There are two well-known environmental account systems:

UN System of Environmental-Economic Accounting SEEA: A satellite account of the System of National Accounts (SNA) that supports decision-making [21]. The SEEA is of benefit to academic researchers, scientists, and agencies providing information that can be used to underpin their decisions [3,6,15,17,19,24]. In this work, we use the UN SEEA system for compiling an inequality satellite account.

NAMEA: In the late 1980s, the Central Bureau of Statistics of the Netherlands developed a system for describing environmental flows in conjunction with the National Accounts. The system, known as the National Accounting Matrix including Environmental Accounts (NAMEA) creates a link between the National Accounts and environmental statistics [5]. NAMEA tables can be used to examine the relationship between environmental pressure and consumption and production patterns [7,13,14,23].

**3.2 Basic definitions of social indicators**

Horn [11] describes social indicators as statistical measures of non-monetary factors that mainly deal with human performance. Gallopin [10] defines the social indicator as a variable that is usually used to describe an attribute of a phenomenon. The OECD [18] describes social indicators as a mirrors that reflect most of our social activities such as health and education. In this regard, the social indicator may be considered as an intermediary that describes the phenomenon.

**3.3 Constructing the satellite and income satellite accounts**

This section is kept brief because the construction principles of global satellite accounts have been determined elsewhere [16].

There are three important steps needed to construct a satellite account: identify databases; prepare constraints data; and create a concordance matrix.

*1. Identify databases*

There are many data sources that provide information about different indicators (such as <http://data.un.org/>). These indicators may have been collected at different times and locations. Sometimes, they are broken down into sub-indices. These sub-indices usually provide more information in a complex system, for example, GHG (indicator) has $\mathrm{CH}_{4}$ as sub-indicator, and $\mathrm{CH}_{4}$ also has fuel as sub-index. In some cases, further disaggregation is needed to provide a deeper understanding of the indicator. These hierarchical levels are meaningful and useful.

*2. Prepare constraints data*

Input-Output tables are often subject to constraints. A constraint is a mathematical condition imposed onto elements of global Multi-Region Input-Output (MRIO) tables. These constraints can be expressed in mathematical equations [16]. Constraints data need to be prepared with names that distinguishes and saves the data according to specific parameters. These parameters are separated by underscore or slash characters to fit the global MRIO system and the output is saved as comma separated files (csv).

*3. Concordances*

We used employment data from the International Labour Organization database (laborsta). These databases are classified by International Standard Industrial Classification ISIC versions (1, 2, 3, and 4). To link between these different versions we prepared a concordance matrix. To reach a high level of disaggregation, we linked the classifications to the Harmonized Commodity Description and Coding System (HS), which is, comprises about 5,000 commodity groups (see [16]).

***Appendix 4:* Further comparisons of Lorenz curves based on measured income and employment distributions and fitted based on Gini index data.**

Below are some examples of Lorenz curves calculated from income and employment quintiles or deciles to determine the Gini index of nations (circles represent the data [1,2,8,9,12] and the lines represent the power function approximation we used (these data populate $I$ and $P$ in section 3.4)).


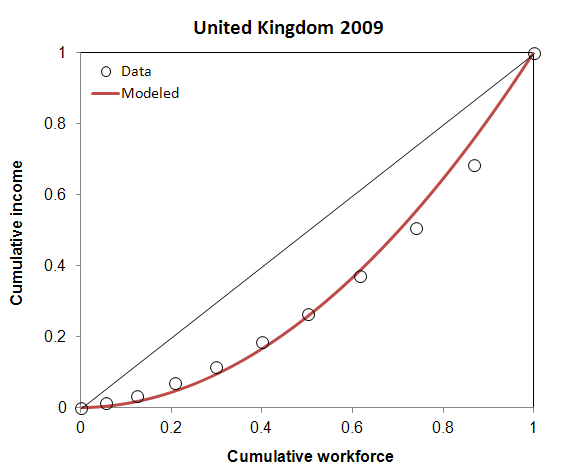

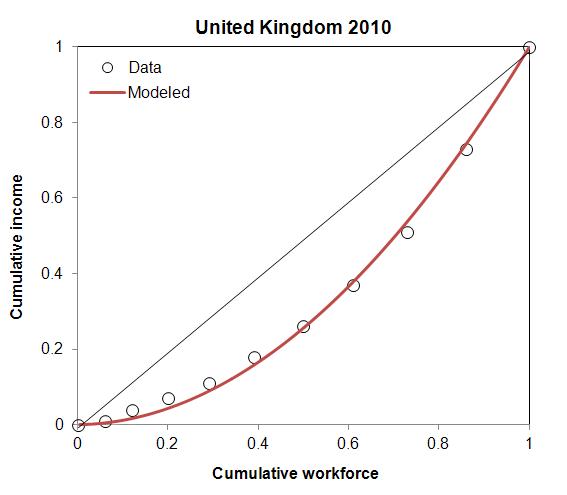


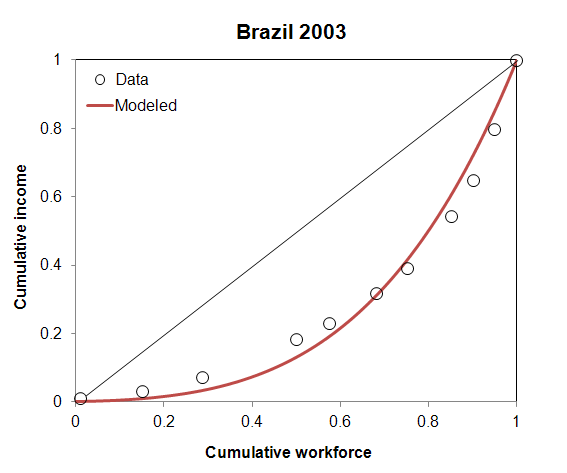

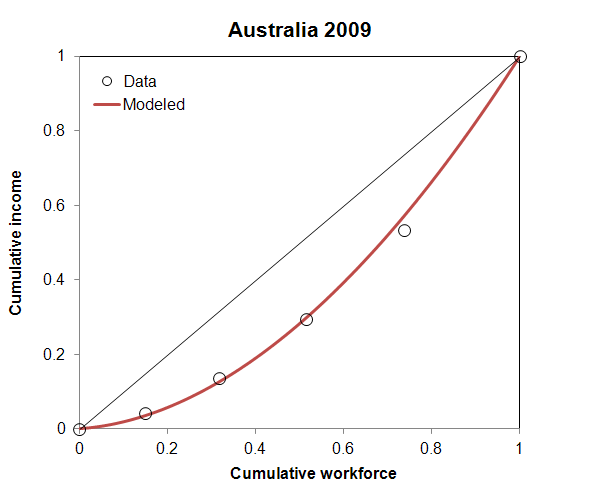

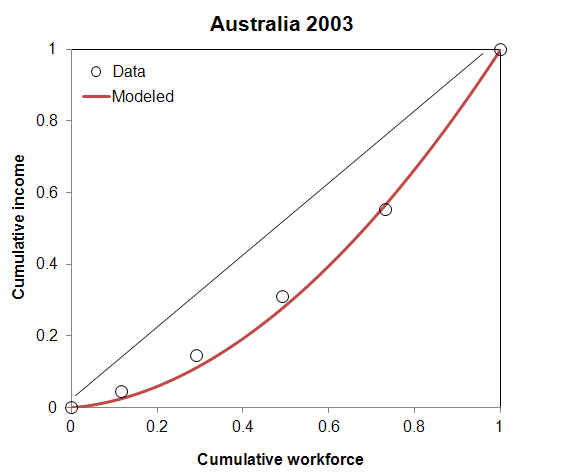


***Appendix 5:* Table S5 the ranking of added countries.**

| Added-countries | Rank |
| --- | --- |
| France | 35 |
| Republic of Korea | 40 |
| United Kingdom | 55 |
| United States | 63 |
| Thailand | 141 |
| China | 154 |

This table shows the ranking of the countries that were added to Fig. 3. Our research covered 187 countries. They were added because of their significant share of the world’s Gross Domestic Product (GDP).

***Appendix 6:* Table S6 additional information that supports Fig. 3 and Table 1 in the manuscript.**

| Country | $\frac{Inequality footprint}{Gini index of domestic workforce}$ | Inequality footprint | Within-country Gini index |
| --- | --- | --- | --- |
| Norway | 1.31 | 0.31 | 0.24 |
| Slovenia | 1.26 | 0.30 | 0.24 |
| Hungary | 1.26 | 0.30 | 0.24 |
| Netherlands | 1.25 | 0.32 | 0.26 |
| Finland | 1.25 | 0.32 | 0.25 |
| Iceland | 1.23 | 0.32 | 0.26 |
| Sweden | 1.23 | 0.30 | 0.24 |
| Japan | 1.19 | 0.34 | 0.29 |
| Canada | 1.14 | 0.34 | 0.30 |
| Austria | 1.14 | 0.30 | 0.26 |
| Switzerland | 1.13 | 0.33 | 0.30 |
| Denmark | 1.12 | 0.30 | 0.27 |
| Australia | 1.09 | 0.34 | 0.31 |
| Germany | 1.09 | 0.32 | 0.29 |
| France | 1.06 | 0.32 | 0.30 |
| Republic of Korea | 1.04 | 0.33 | 0.32 |
| United Kingdom | 0.97 | 0.32 | 0.33 |
| United States | 0.95 | 0.34 | 0.36 |
| Thailand | 0.78 | 0.31 | 0.40 |
| China | 0.75 | 0.32 | 0.42 |
| Philippines | 0.75 | 0.32 | 0.43 |
| Mexico | 0.72 | 0.35 | 0.48 |
| Malaysia | 0.70 | 0.33 | 0.46 |
| Brazil | 0.64 | 0.35 | 0.55 |
| Russian Federation | 0.57 | 0.27 | 0.46 |
| South Africa | 0.53 | 0.34 | 0.63 |

This table supports the detail of Fig. 3 and Table 1, it contains: ratio of inequality footprint to within-country Gini index, inequality footprint (populate $\overline{\gamma}_{X}$in section 3.5), and within-country Gini index (populate$\gamma$* *in section 3.4).

***Appendix 7:***


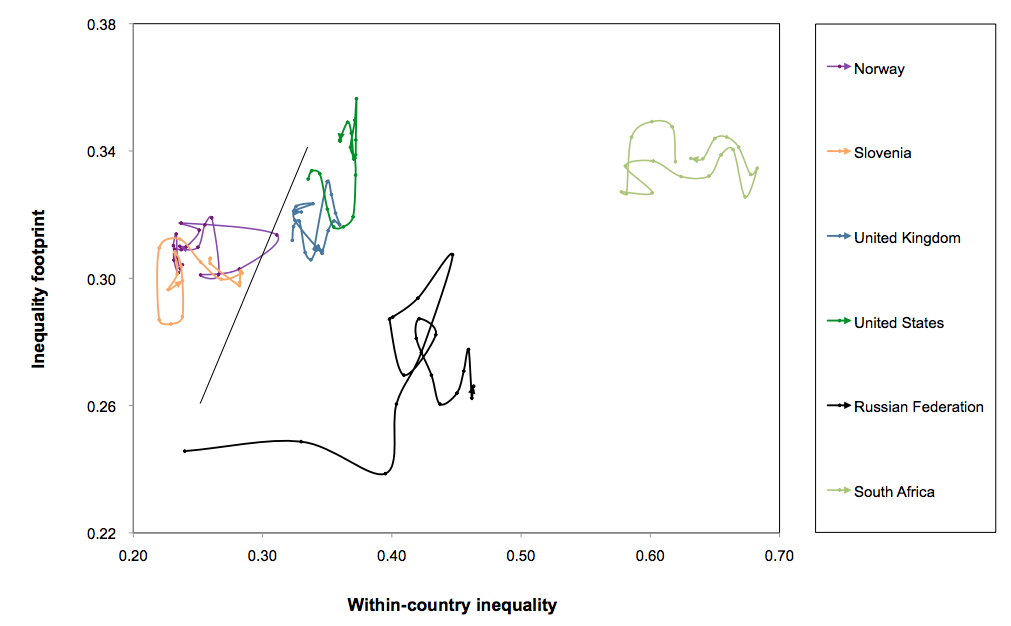


**FIGURE S2**

Example of time series of within-country Gini index (populate$\gamma$*  *in section 3.4) against inequality footprint (populate$\overline{\gamma}_{X}$in section 3.5) spanning 1990-2010 for the top and bottom two countries of Fig. 3 and including United Kingdom and United States (each year is represented by a dot point and 2010 is represented by the arrowhead).

| **Year** | **within country Gini index** | **Inequality footprint** | **within country Gini index** | **Inequality footprint** | **within country Gini index** | **Inequality footprint** | **within country Gini index** | **Inequality footprint** | **within country Gini index** | **Inequality footprint** | **within country Gini index** | **Inequality footprint** |
| --- | --- | --- | --- | --- | --- | --- | --- | --- | --- | --- | --- | --- |
|  | Norway | Norway | Slovenia | Slovenia | United States | United States | Russian Federation | Russian Federation | South Africa | South Africa | United Kingdom | United Kingdom |
| 1990 | 0.23 | 0.31 | 0.26 | 0.31 | 0.34 | 0.33 | 0.24 | 0.25 | 0.62 | 0.34 | 0.32 | 0.31 |
| 1991 | 0.23 | 0.31 | 0.26 | 0.31 | 0.34 | 0.33 | 0.33 | 0.25 | 0.62 | 0.35 | 0.32 | 0.32 |
| 1992 | 0.23 | 0.31 | 0.26 | 0.30 | 0.34 | 0.33 | 0.40 | 0.24 | 0.60 | 0.35 | 0.33 | 0.32 |
| 1993 | 0.23 | 0.31 | 0.28 | 0.30 | 0.35 | 0.32 | 0.40 | 0.26 | 0.59 | 0.34 | 0.33 | 0.31 |
| 1994 | 0.24 | 0.30 | 0.28 | 0.30 | 0.36 | 0.32 | 0.42 | 0.28 | 0.58 | 0.33 | 0.34 | 0.31 |
| 1995 | 0.24 | 0.30 | 0.28 | 0.30 | 0.36 | 0.32 | 0.45 | 0.31 | 0.58 | 0.33 | 0.34 | 0.31 |
| 1996 | 0.23 | 0.30 | 0.28 | 0.30 | 0.37 | 0.32 | 0.42 | 0.29 | 0.60 | 0.33 | 0.35 | 0.31 |
| 1997 | 0.23 | 0.31 | 0.28 | 0.30 | 0.37 | 0.33 | 0.40 | 0.29 | 0.58 | 0.34 | 0.35 | 0.31 |
| 1998 | 0.23 | 0.31 | 0.28 | 0.30 | 0.37 | 0.34 | 0.40 | 0.29 | 0.60 | 0.34 | 0.36 | 0.32 |
| 1999 | 0.24 | 0.31 | 0.27 | 0.30 | 0.37 | 0.34 | 0.41 | 0.27 | 0.62 | 0.33 | 0.36 | 0.32 |
| 2000 | 0.25 | 0.31 | 0.25 | 0.31 | 0.37 | 0.34 | 0.43 | 0.28 | 0.65 | 0.33 | 0.36 | 0.32 |
| 2001 | 0.26 | 0.32 | 0.24 | 0.31 | 0.37 | 0.35 | 0.42 | 0.29 | 0.65 | 0.34 | 0.35 | 0.33 |
| 2002 | 0.26 | 0.32 | 0.22 | 0.31 | 0.37 | 0.36 | 0.42 | 0.28 | 0.66 | 0.34 | 0.35 | 0.33 |
| 2003 | 0.27 | 0.30 | 0.22 | 0.29 | 0.37 | 0.34 | 0.43 | 0.27 | 0.67 | 0.33 | 0.34 | 0.31 |
| 2004 | 0.25 | 0.30 | 0.23 | 0.29 | 0.37 | 0.34 | 0.44 | 0.26 | 0.68 | 0.33 | 0.34 | 0.31 |
| 2005 | 0.28 | 0.30 | 0.24 | 0.29 | 0.37 | 0.34 | 0.45 | 0.26 | 0.68 | 0.33 | 0.35 | 0.31 |
| 2006 | 0.31 | 0.31 | 0.24 | 0.30 | 0.37 | 0.35 | 0.46 | 0.27 | 0.67 | 0.34 | 0.33 | 0.32 |
| 2007 | 0.24 | 0.32 | 0.23 | 0.31 | 0.37 | 0.35 | 0.46 | 0.28 | 0.66 | 0.34 | 0.33 | 0.32 |
| 2008 | 0.25 | 0.32 | 0.23 | 0.30 | 0.36 | 0.34 | 0.46 | 0.26 | 0.65 | 0.34 | 0.34 | 0.32 |
| 2009 | 0.24 | 0.31 | 0.23 | 0.30 | 0.36 | 0.34 | 0.46 | 0.27 | 0.64 | 0.34 | 0.32 | 0.32 |
| 2010 | 0.24 | 0.31 | 0.24 | 0.30 | 0.36 | 0.34 | 0.46 | 0.27 | 0.63 | 0.34 | 0.33 | 0.32 |

***Appendix 7:* Table S7-1 additional information that supports the figure S2.**

***Appendix 7:***

Other examples


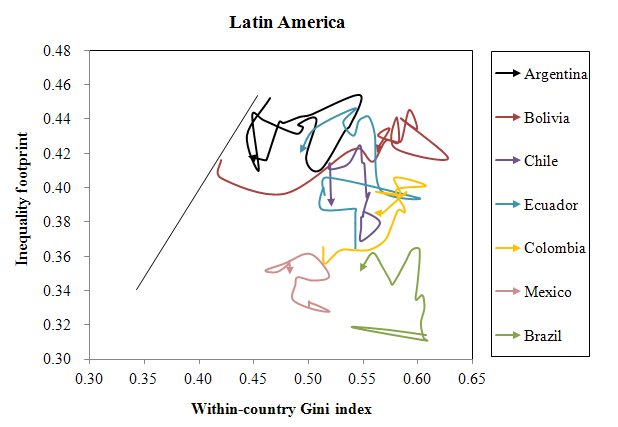


**FIGURE S3**

***Appendix 7:* Table S7-2 additional information that supports the figure S3 (Latin America).**

| **Year** | **within country Gini index** | **Inequality footprint** | **within country Gini index** | **Inequality footprint** | **within country Gini index** | **Inequality footprint** | **within country Gini index** | **Inequality footprint** | **within country Gini index** | **Inequality footprint** | **within country Gini index** | **Inequality footprint** | **within country Gini index** | **Inequality footprint** |
| --- | --- | --- | --- | --- | --- | --- | --- | --- | --- | --- | --- | --- | --- | --- |
|  | Argentina | Argentina | Bolivia | Bolivia | Chile | Chile | Ecuador | Ecuador | Colombia | Colombia | Mexico | Mexico | Brazil | Brazil |
| 1990 | 0.47 | 0.45 | 0.42 | 0.42 | 0.55 | 0.38 | 0.54 | 0.38 | 0.51 | 0.37 | 0.50 | 0.33 | 0.61 | 0.31 |
| 1991 | 0.44 | 0.43 | 0.42 | 0.41 | 0.55 | 0.38 | 0.54 | 0.38 | 0.51 | 0.36 | 0.50 | 0.33 | 0.57 | 0.32 |
| 1992 | 0.45 | 0.41 | 0.48 | 0.40 | 0.55 | 0.37 | 0.54 | 0.37 | 0.51 | 0.36 | 0.50 | 0.33 | 0.54 | 0.32 |
| 1993 | 0.45 | 0.44 | 0.54 | 0.42 | 0.56 | 0.38 | 0.54 | 0.39 | 0.53 | 0.36 | 0.51 | 0.33 | 0.61 | 0.31 |
| 1994 | 0.46 | 0.44 | 0.55 | 0.42 | 0.55 | 0.39 | 0.54 | 0.39 | 0.54 | 0.36 | 0.52 | 0.33 | 0.61 | 0.31 |
| 1995 | 0.49 | 0.43 | 0.56 | 0.42 | 0.55 | 0.38 | 0.51 | 0.39 | 0.56 | 0.36 | 0.50 | 0.33 | 0.60 | 0.32 |
| 1996 | 0.50 | 0.44 | 0.57 | 0.43 | 0.55 | 0.38 | 0.51 | 0.40 | 0.57 | 0.37 | 0.49 | 0.33 | 0.61 | 0.33 |
| 1997 | 0.49 | 0.44 | 0.58 | 0.43 | 0.55 | 0.39 | 0.52 | 0.40 | 0.58 | 0.38 | 0.49 | 0.34 | 0.61 | 0.33 |
| 1998 | 0.51 | 0.44 | 0.58 | 0.44 | 0.56 | 0.40 | 0.52 | 0.41 | 0.58 | 0.39 | 0.49 | 0.35 | 0.60 | 0.34 |
| 1999 | 0.50 | 0.42 | 0.58 | 0.42 | 0.55 | 0.40 | 0.60 | 0.39 | 0.59 | 0.39 | 0.50 | 0.35 | 0.60 | 0.33 |
| 2000 | 0.51 | 0.41 | 0.63 | 0.42 | 0.55 | 0.39 | 0.57 | 0.40 | 0.59 | 0.39 | 0.52 | 0.35 | 0.60 | 0.35 |
| 2001 | 0.53 | 0.43 | 0.59 | 0.44 | 0.55 | 0.41 | 0.56 | 0.43 | 0.58 | 0.40 | 0.51 | 0.36 | 0.60 | 0.36 |
| 2002 | 0.54 | 0.44 | 0.60 | 0.43 | 0.55 | 0.41 | 0.56 | 0.44 | 0.61 | 0.40 | 0.50 | 0.36 | 0.59 | 0.36 |
| 2003 | 0.55 | 0.45 | 0.59 | 0.45 | 0.55 | 0.43 | 0.55 | 0.44 | 0.58 | 0.41 | 0.48 | 0.36 | 0.59 | 0.36 |
| 2004 | 0.50 | 0.44 | 0.59 | 0.43 | 0.54 | 0.41 | 0.55 | 0.44 | 0.58 | 0.40 | 0.46 | 0.35 | 0.58 | 0.34 |
| 2005 | 0.49 | 0.44 | 0.58 | 0.43 | 0.53 | 0.41 | 0.54 | 0.43 | 0.56 | 0.40 | 0.47 | 0.35 | 0.57 | 0.35 |
| 2006 | 0.48 | 0.44 | 0.56 | 0.43 | 0.52 | 0.41 | 0.53 | 0.44 | 0.59 | 0.40 | 0.48 | 0.36 | 0.57 | 0.35 |
| 2007 | 0.47 | 0.44 | 0.57 | 0.43 | 0.52 | 0.41 | 0.54 | 0.45 | 0.59 | 0.40 | 0.48 | 0.36 | 0.56 | 0.36 |
| 2008 | 0.46 | 0.42 | 0.56 | 0.42 | 0.52 | 0.40 | 0.51 | 0.43 | 0.57 | 0.39 | 0.48 | 0.35 | 0.55 | 0.36 |
| 2009 | 0.46 | 0.42 | 0.56 | 0.42 | 0.52 | 0.39 | 0.49 | 0.42 | 0.57 | 0.39 | 0.48 | 0.35 | 0.55 | 0.35 |
| 2010 | 0.44 | 0.42 | 0.56 | 0.42 | 0.52 | 0.39 | 0.49 | 0.42 | 0.56 | 0.39 | 0.48 | 0.35 | 0.55 | 0.35 |

***Appendix 7:***

Other examples

**
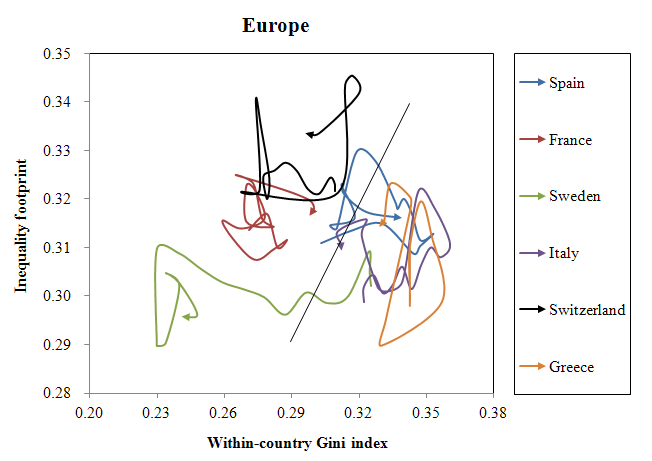
**

**FIGURE S4**

***Appendix 7:* Table S7-3 additional information that supports the figure S4 (Europe).**

| **Year** | **within country Gini index** | **Inequality footprint** | **within country Gini index** | **Inequality footprint** | **within country Gini index** | **Inequality footprint** | **within country Gini index** | **Inequality footprint** | **within country Gini index** | **Inequality footprint** | **within country Gini index** | **Inequality footprint** |
| --- | --- | --- | --- | --- | --- | --- | --- | --- | --- | --- | --- | --- |
|  | Spain | Spain | France | France | Sweden | Sweden | Italy | Italy | Switzerland | Switzerland | Greece | Greece |
| 1990 | 0.30 | 0.31 | 0.27 | 0.31 | 0.33 | 0.30 | 0.32 | 0.30 | 0.31 | 0.32 | 0.34 | 0.30 |
| 1991 | 0.32 | 0.31 | 0.28 | 0.32 | 0.32 | 0.30 | 0.32 | 0.30 | 0.31 | 0.32 | 0.34 | 0.30 |
| 1992 | 0.33 | 0.31 | 0.28 | 0.32 | 0.32 | 0.31 | 0.33 | 0.30 | 0.31 | 0.32 | 0.34 | 0.31 |
| 1993 | 0.34 | 0.31 | 0.28 | 0.31 | 0.32 | 0.30 | 0.33 | 0.30 | 0.30 | 0.32 | 0.34 | 0.30 |
| 1994 | 0.35 | 0.31 | 0.29 | 0.31 | 0.31 | 0.30 | 0.33 | 0.30 | 0.30 | 0.32 | 0.34 | 0.30 |
| 1995 | 0.35 | 0.31 | 0.28 | 0.31 | 0.30 | 0.30 | 0.34 | 0.31 | 0.29 | 0.33 | 0.34 | 0.30 |
| 1996 | 0.35 | 0.31 | 0.27 | 0.31 | 0.29 | 0.30 | 0.34 | 0.30 | 0.29 | 0.33 | 0.34 | 0.31 |
| 1997 | 0.34 | 0.32 | 0.26 | 0.31 | 0.28 | 0.30 | 0.35 | 0.31 | 0.28 | 0.33 | 0.34 | 0.31 |
| 1998 | 0.34 | 0.32 | 0.26 | 0.32 | 0.27 | 0.30 | 0.35 | 0.31 | 0.28 | 0.32 | 0.34 | 0.31 |
| 1999 | 0.34 | 0.32 | 0.27 | 0.31 | 0.26 | 0.30 | 0.36 | 0.31 | 0.28 | 0.32 | 0.34 | 0.30 |
| 2000 | 0.34 | 0.32 | 0.28 | 0.32 | 0.25 | 0.31 | 0.36 | 0.31 | 0.28 | 0.32 | 0.34 | 0.31 |
| 2001 | 0.33 | 0.33 | 0.27 | 0.32 | 0.24 | 0.31 | 0.35 | 0.32 | 0.28 | 0.33 | 0.35 | 0.32 |
| 2002 | 0.32 | 0.33 | 0.27 | 0.32 | 0.23 | 0.31 | 0.35 | 0.32 | 0.27 | 0.34 | 0.35 | 0.31 |
| 2003 | 0.31 | 0.31 | 0.27 | 0.32 | 0.23 | 0.29 | 0.34 | 0.30 | 0.28 | 0.32 | 0.36 | 0.30 |
| 2004 | 0.31 | 0.31 | 0.28 | 0.31 | 0.23 | 0.29 | 0.33 | 0.30 | 0.27 | 0.32 | 0.33 | 0.29 |
| 2005 | 0.32 | 0.32 | 0.28 | 0.32 | 0.23 | 0.29 | 0.33 | 0.30 | 0.31 | 0.32 | 0.33 | 0.30 |
| 2006 | 0.31 | 0.32 | 0.27 | 0.32 | 0.24 | 0.30 | 0.32 | 0.31 | 0.31 | 0.34 | 0.34 | 0.32 |
| 2007 | 0.31 | 0.32 | 0.27 | 0.32 | 0.23 | 0.30 | 0.32 | 0.32 | 0.32 | 0.35 | 0.34 | 0.32 |
| 2008 | 0.31 | 0.32 | 0.30 | 0.32 | 0.24 | 0.30 | 0.31 | 0.31 | 0.32 | 0.34 | 0.33 | 0.32 |
| 2009 | 0.32 | 0.32 | 0.30 | 0.32 | 0.25 | 0.30 | 0.32 | 0.31 | 0.30 | 0.33 | 0.33 | 0.31 |
| 2010 | 0.34 | 0.32 | 0.30 | 0.32 | 0.24 | 0.30 | 0.31 | 0.31 | 0.30 | 0.33 | 0.33 | 0.31 |

**REFERENCES**

1. ABS (2010) Household Expenditure Survey - Detailed Expenditure Items. ABS Catalogue No. 6530.0, (Canberra, Australia, Australian Bureau of Statistics).
2. ABS (2004) Household Expenditure Survey - Detailed Expenditure Items. ABS Catalogue No. 6530.0, (Canberra, Australia, Australian Bureau of Statistics).
3. Bartelmus P (2007) SEEA-2003 Accounting for sustainable development?. Ecol. Econ. 61, 613-616.
4. Costantini V, M Mazzanti, A Montini (2011) Hybrid Economic-Environmental Accounts. (Taylor and Francis).
5. de Haan M, SJ Keuning (1996) Taking the environment into account: the NAMEA approach. Review of Income and Wealth 42, 131-148.
6. Dietz S, E Neumayer (2007) Weak and strong sustainability in the SEEA: Concepts and measurement. Ecological Economics 61, 617-626.
7. EC (2001) NAMEAs for air emissions: Results of pilot studies. (Luxembourg, European Communities).
8. Family Spending (2011) Edition Released: 29 November 2011. (UK office for national statistics). Accessed 25 January 2013.
9. Family Spending (2010) Edition Released: 30 November 2010. (UK office for national statistics). Accessed 25 January 2013.
10. Gallopin G (1997) Indicators and their use: information for decision making. In: Moldan, B., Billharz S (Eds.) Sustainability Indicators. (Report on the Project on Indicators of Sustainable Development. Wiley, Chicheste).
11. Horn V (1993) Statistical indicators for the economic & social sciences. (Cambridge, UK, Cambridge University Press).
12. IBGE (2010) Pesquisa de Orçamento Familiares. (Rio de Janeiro, Brazil, Instituto Brasileiro de Geografia e Estatística, Ministério da Fazenda).
13. Ike T (1999) A Japanese NAMEA. Structural Change and Economic Dynamics 10, 123-149.
14. Keuning SJ, J. van Dalen, M de Haan (1999) The Netherlands 'NAMEA'; presentation, usage and future extensions. Structural Change and Economic Dynamics 10, 15-37.
15. Lange GM (2007) Environmental accounting: Introducing the SEEA-2003. Ecol. Econ. 61, 589-591.
16. Lenzen M, K Kanemoto, D Moran, A Geschke (2012) Mapping the structure of the world economy. Environ. Sci. Technol. 46, 8374–8381, <http://dx.doi.org/10.1021/es300171x>.
17. Lenzen M, J Murray (2011) Accounting for Carbon Flows: Comparing the Principles of the UNFCCC and the SEEA. Society and Natural Resources 24, 1216-1227.
18. OECD (2009) Interpreting OECD Social Indicator in Society at a Glance 2009: (OECD Social Indicators. OECD Publishing. <http://dx.doi.org/10.1787/soc_glance-2008-4-en>)
19. Smith R (2006) Development of the SEEA 2003 and its implementation. Ecol. Econ. 61, 592-599.
20. Solt Frederick (2009) Standardizing the World Income Inequality Database. Social Science Quarterly 90 (2), 231-242. SWIID Version 3.1, December 2011.
21. UNSD (2003) Handbook of National Accounting: Integrated Environmental and Economic Accounting (SEEA 2003). (New York, USA, <http://unstats.un.org/unsd/envAccounting/seea2003.pdf>, United Nations Statistics Division).
22. UNSD (2009) System of National Accounts 1993. Internet site unstats.un.org/unsd/sna1993/toctop.asp (New York, USA, United Nations Statistics Division).
23. Vaze P (1999) A NAMEA for the UK. Structural Change and Economic Dynamics 10, 99-121.
24. Walker BH, L Pearson (2007) A resilience perspective of the SEEA. Ecol. Econ. 61, 708-715.
